# Lipopolysaccharide Pre-conditioning Attenuates Pro-inflammatory Responses and Promotes Cytoprotective Effect in Differentiated PC12 Cell Lines *via* Pre-activation of Toll-Like Receptor-4 Signaling Pathway Leading to the Inhibition of Caspase-3/Nuclear Factor-κappa B Pathway

**DOI:** 10.3389/fncel.2020.598453

**Published:** 2021-01-22

**Authors:** Pushpa Gandi Sangaran, Zaridatul Aini Ibrahim, Zamri Chik, Zahurin Mohamed, Abolhassan Ahmadiani

**Affiliations:** ^1^Department of Pharmacology, Faculty of Medicine, University of Malaya, Kuala Lumpur, Malaysia; ^2^Department of Pharmacology, School of Medicine, Shahid Beheshti University of Medical Sciences, Evin, Tehran, Iran

**Keywords:** Neuroinflammation, Lipopolysaccharide, Toll-like Receptor 4, Pre-conditioning, cytoprotective, apoptosis, caspase, Nuclear Factor Kappa B

## Abstract

Lipopolysacharide (LPS) pre-conditioning (PC), has been shown to exert protective effects against cytotoxic effects. Therefore, we hypothesized, the tolerance produced by LPS PC will be resulted by the alterations and modifications in gene and protein expression. With reference to the results of MTT assays, AO/PI staining, and Annexin V-FITC analyses of LPS concentration (0.7815–50 μg/mL) and time-dependent (12–72 h) experiments, the pre-exposure to 3 μg/mL LPS for 12 h protected the differentiated PC12 cells against 0.75 mg/mL LPS apoptotic concentration. LPS-treated cells secreted more inflammatory cytokines like IL-1α, IL-1β, IL-2, IL-3, IL-4, IL-6, IL-17, IFN-γ, and TNF-α than LPS-PC cells. The production of inflammatory mediators ROS and NO was also higher in the LPS-induced cells compared to LPS-PC cells. Conversely, anti-inflammatory cytokines (like IL-10, IL-13, CNTF, and IL-1Ra) were upregulated in the LPS-PC cells but not in the LPS-induced cells. Meanwhile, the LPS initiated caspase-8 which in turn activates effector caspase 3/7. When the activities of caspases in the LPS-induced cells were inhibited using z-VADfmk and z-DEVDfmk, the expressions of c-MYC and Hsp70 were increased, but p53 was reduced. The potential molecules associated with protective and destructive effect was measured by RT^2^ Profiler PCR array to elucidate the signaling pathways and suggested inhibition NF-κB/caspase-3 signaling pathway regulates the cytoprotective genes and proto-oncogenes. In conclusion, this study provides a basis for future research to better understand the molecular mechanism underlying LPS pre-conditioning /TLR4 pre-activation and its functional role in offering cytoprotective response in neuronal environment.

## Introduction

Brain diseases remain a clinical problem as we have incomplete understanding of their pathogenesis. Indeed, cerebral ischemia after cardiac arrest, focal occlusion of the brain vessel, traumatic brain injury and ischemia brain damage after cardiac or brain surgery causes adult disability and death of millions of people worldwide (Feigin et al., 2010). Moreover, the brain has long been deliberated as an immune-privileged site because of the presence of blood brain barrier (BBB) and the deficiency of the lymphatic system is also capable of inflammatory response. In contrast, invading pathogens, trauma, and infections trigger glial cells to secrete reactive oxygen species (ROS)/ reactive nitrogen species (RNS) inflammatory mediators and neurotoxic free radicals, which may contribute to brain inflammation or neuroinflammation. Emerging evidence indicates that neuroinflammation is closely associated with neurodegenerative diseases. Therefore, regulation of neuroinflammation is essential to maintain the environment in nervous tissue and prevent the onset of neurodegenerative diseases.

Even though many pharmacological drugs appear promising in animal models, not many pharmacological agents have been successfully translated to human brain injury and other neurodegenerative diseases. This could be due to the difficulty of the drugs to pass through BBB, that is highly selective and semipermeable and restricts the movement of drug molecules into the brain (Loane and Faden, 2010). However, this is a complex process how the drugs pass through BBB and the reasons are unclear, yet to be proven. In spite of many promising experimental studies, thus far, there is no effective treatment to eliminate the pathological complications of the brain. These could be explained that if acute neuroinflammation could not be treated to maintain the homeostatic condition, then the chronic complications would cause a complex of pathological processes which includes massive inflammatory response, ischemia, apoptosis, oxidative stress, and tissue necrosis.

Therefore, researchers have focused on triggering the inflammatory response of the immune system natural adaptive process, which confers neuroprotection and limits chronic complications through certain signaling pathways. As a result, there has been much interest in research regarding pre-conditioning, which could be used as a therapeutic technique by inducing tolerance and protection against stressors. Pre-conditioning is a procedure by which a noxious stimulus near to but below the threshold of damage is applied to the tissue, thereby inducing tolerance or resistance (Dirnagl et al., 2009).

Take note, pre-conditioning in neuropathology has been previously researched in different types of animal models and diseases such as stroke, cerebral ischemia, Alzheimer's disease, Parkinson's disease, multiple sclerosis, traumatic brain injury, and other neurodegenerative diseases (Stetler et al., 2014). There are various types of pre-conditioning including ischemic pre-conditioning, oxygen pre-conditioning, temperature pre-conditioning, pharmacological pre-conditioning, neuroinflammatory agent pre-conditioning, systemic stress pre-conditioning, and subcellular stress pre-conditioning. In such studies, the animal models are pre-conditioned with various endogenous or exogenous stimuli to adapt to the injury and enhance the ability of the cells to survive (Dirnagl et al., 2009).

In addition to the type of pre-conditioned stimuli, time window is also taken into consideration to provide protection. It is possible that the time window could increase the tolerance to the stimuli to confer the protection. The protective effects have two windows of protection: (i) classical or early pre-conditioning, which lasts for 4–6 h and (ii) late phase or delayed pre-conditioning, which begins at 24 h and lasts for 72 h (Ramzy et al., 2006). A study by Siddiq et al. (2008) showed that repetitive hypoxic pre-conditioning is associated with time window and is related to immune tolerance, inducing neuroprotection in the retina and lasting for many weeks. Rapid pre-conditioning seems to be practical in clinical practices and could be applied therapeutically in the same settings of chronic complications. Therefore, it is important to induce protection against lethal injury at an ultra low dose or concentration, depending on the time frame (Doyle et al., 2008).

Most pre-conditioning studies are short with limited periods of survival. Moreover, the strategy of inducing immunological tolerance in CNS is complicated as the particular drug have difficulties to pass through BBB where it has to compromise with BBB. But, if the CNS immune response is completely suppressed, it may not provide the protective mechanism. Other than that, animal models may not respond to a particular drug as humans do due to genetic variations (pharmacogenomics) and physiological differences. Therefore, *in vitro* models could be useful to expose the cells directly to the drug and manipulate the environmental factors to modulate cell response (Jucker, 2010). Pre-conditioning with sublethal dose or concentration preserves cell viability against apoptosis or lethal injury. Thus far, pre-conditioning has been studied in HEK 293 cells derived from human embryonic kidney, HIT-T15 cells from Syrian hamster pancreatic islets, and C2C12 cells from mouse skeletal muscle (Liu et al., 1996).

On the other hand, LPS is widely used in experimental models to activate the innate immunity which induces microglial activation and neuroinflammation (Rosenzweig et al., 2004). In the 16th century, Paracelsus, a toxicologist, posited that “the dose makes the poison.” He found that administration of low LPS dose induces a protective state against a higher dose of LPS which has the tolerance state. This phenomenon is known as “LPS pre-conditioning,” where pre-stimulation to a low concentration of LPS could prevent the cells from undergoing apoptosis in response to subsequent stimulation with LPS at higher concentration, suggesting a role for TLR4 pre-activation in the signaling pathway of LPS-induced neuroprotection (Stetler et al., 2014). Moreover, researchers have found that LPS pre-conditioning attenuates apoptosis, protects against cytotoxic effects in stroke, facilitates microglial activation after spinal cord injury, confers neuroprotection against axonal injury, mediates tolerance to ischemic injury, and reduces post-injury, gliosis, and other neuropathology diseases (Hayakawa et al., 2014).

Even though it is well known that apoptosis contributes to neuronal cell death in various neurodegenerative diseases with the activation of caspases (Shi, 2004), in contrast, the inhibition of caspase activity provides neuroprotection. Several studies have suggested that apoptosis inhibition could be a therapeutic option in acute and chronic neurodegenerative diseases (Wellington and Hayden, 2000). As caspases are the downstream molecules of TLR4 signaling pathway, it would be of interest to investigate if LPS pre-conditioning is associated with caspases and NF-κB inhibition, which provide cytoprotection. The findings by Rosenzweig *et al*. suggested that the protective state is known as pre-conditioning phenomenon or tolerance, which modulates the inflammatory response and confers neuroprotection (Rosenzweig et al., 2007). In contrast, it is also possible that the inhibition of caspase and NF-κB could promote oncogene expressions (Karin and Lin, 2002).

However, to date, to the best of our knowledge, there is poor understanding regarding *in vitro* research on LPS pre-conditioning. On top of that, the way in which low concentration of LPS binds the TLR4 signaling pathway and induces the cytoprotective response is also poorly understood. To achieve the understanding of the cellular and molecular mechanisms of LPS pre-conditioning, we demonstrated that LPS pre-conditioning confers cytoprotection against LPS-induced apoptosis.

Therefore, in the present study, LPS pre-conditioning will be performed *in vitro* with the hypothesis that the caspase-3/NF-κB signaling pathway could have a dual role of protecting the cells against apoptosis or promoting oncogene expression. The details linking the caspases activation and inhibition, which are linked to the apoptosis mechanism and oncogene expression, will be discussed further in this research.

For that reason, we used differentiated PC12 cells as a model to study LPS pre-conditioning and investigate how it confers protection against apoptosis. Yet, thus far, no research has been done on LPS pre-conditioning in PC12 cell line. Therefore, we will explore the underlying molecular mechanisms involved in LPS pre-conditioning in differentiated PC12 cells, which confer protection against LPS-induced apoptosis mechanism in detail. Other than that, proper pre-conditioning setup is needed especially on the important principles such as concentration or dose along with the time frame to provide protection. In order to achieve that, the goals of our research were (1) to optimize the concentration and the exposure duration of LPS pre-conditioning that will induce protection against apoptosis in differentiated PC12 cells, (2) to investigate the effect of LPS pre-conditioning on the expression and/or production of cytokines, chemokines, reactive oxygen species (ROS), nitric oxide (NO), as well as pro-apoptotic proteins and genes, and (3) to elucidate the downstream signaling pathway involved in LPS-induced responses in *in vitro* differentiated PC12 cells.

## Materials and Methods

### PC12 Cell Culture and Differentiation

Pheochromocytoma (PC12) cells, a cell line derived from rat adrenal medulla, were obtained from the Pasteur Institute of Iran (Tehran, Iran). The cells were grown in Dulbecco's Modified Eagle's Medium (DMEM) supplemented with 1000 mg/L glucose, pyruvate, 3.7 g NaHCO_3_/L, 10% heat-inactivated horse serum, and 5% fetal bovine serum. Nerve growth factor (NGF) (50 ng/mL) was added to the medium every other day up to 6 days of culture period to induce neuronal phenotype. The cultures were maintained at 37°C in humidified 5% CO_2_ atmosphere.

PC12 cell line was chosen because it has been demonstrated to be a well-established model for studying the NGF network of signal transduction pathways in neuronal cells (Khodagholi et al., 2012). However, adding NGF to the culture media will end the proliferation of the cells and extend the neurites, which are either axons or dendrites (Greene, 1977). Indeed, many researchers have used PC12 to study neurite outgrowth, toxicity, and the protective effect (Radio and Mundy, 2008).

### Passaging PC12 Cells (Accutase)

Once the PC12 cells reached 80% confluency, the media was removed and the cells were rinsed with PBS thrice. Then, Accutase was added to detach the cells, depending on the volume of cells for 10 min. To stop Accutase action, double the volume of media was added. The suspension was transferred into a Falcon tube and centrifuged at 1,500 rpm for 5 min. The supernatant was discarded and the pellet was resuspended in new complete media. Finally, the cells were seeded for subsequent experiments at their third passage.

### Counting the Cells

The PC12 cells were counted using a Neubauer hemocytometer. First, the hemocytometer was cleaned with 70% ethanol. Then, 10 μL of cells were diluted with 990 μL of trypan blue. Next, 10 μL of the sample was pipetted onto the hemocytometer and the cells were counted from the four quadrants of hemocytometer. Ninety percent of the cells were viable upon determination by trypan blue.

## Preparation of LPS to Induce Differentiated PC12 Cells

### Experimental Groups With LPS Stimulation

**Scheme 1:**
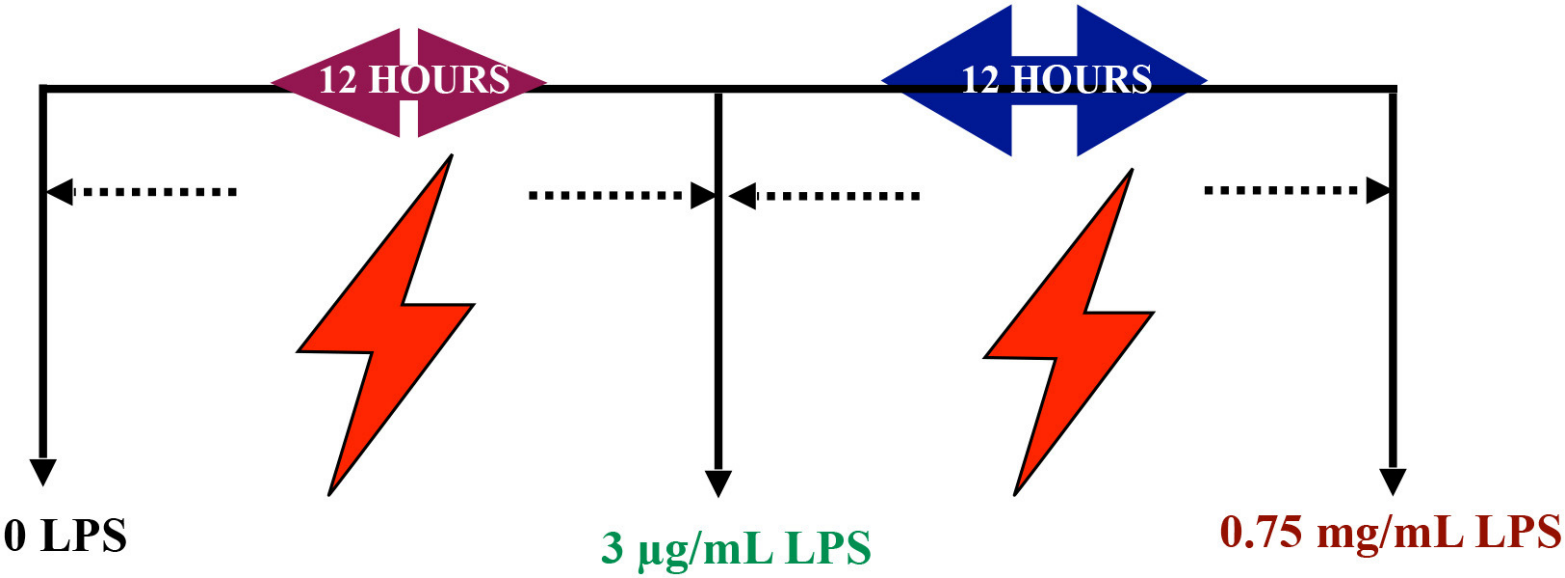
Schematic representation of the experimental timescale.

*In vitro* differentiated PC12 cells were treated with serial range of concentrations 0.7815, 1.563, 3.125, 6.25, 12.5, 25, and 50 μg/mL LPS for 12, 24, 48, and 72 h to optimize the appropriate pre-conditioning concentration. Based on these results, 85% of the cell viability concentration were chosen for subsequent experiments. Next, the differentiated PC12 cells were treated with LPS at pre-conditioning concentrations 3, 6, 9, and 12 μg/mL LPS for 12, 24, 48, and 72 h with 0.75 mg/mL LPS at higher concentration for 12, 24, 48, and 72 h. Then, the differentiated PC12 cells were assigned to four main groups. The optimized concentration of LPS from *Escherichia coli* O55:B5 (Sigma Aldrich, USA) was used to induce differentiated PC12 cells as follows: (1) Control group: Differentiated PC12 cells were incubated in DMEM solution, (2) LPS-induced cells: Differentiated PC12 cells were induced with 0.75 mg/mL LPS for 24 h, and (3) LPS-pre-conditioned cells: Differentiated PC12 cells were pre-treated with 3 μg/mL LPS for 12 h and post-incubated with 0.75 mg/mL LPS for 12 h as mentioned in schematic figure, (4) caspase inhibition group: the differentiated PC12 cells were incubated in DMEM in the presence of Z-VAD-FMK (pan-caspase inhibitor) and Z-DEVD-FMK (selective caspase-3 inhibitor) for 3 h and followed by induction with 0.75 mg/mL LPS (Scheme 1).

### Cell Viability Assay

To obtain the cell viability, the differentiated PC12 cells were seeded in 96-well culture plates at ~5 × 10^3^ cells/well and maintained in a CO_2_ incubator at 37°C overnight. The cells were treated with serial range of concentrations (0.7815, 1.563, 3.125, 6.25, 12.5, 25, and 50 μg/mL) LPS for 12, 24, 48, and 72 h to optimize the appropriate pre-conditioning concentration. Based on these results, 85% of the cell viability concentration were chosen for subsequent experiments. Then, cells were induced with LPS at pre-conditioning concentrations (3, 6, 9, and 12 μg/mL) for 12 h and post-incubated with 0.75 mg/mL LPS for 12, 24, 48, and 72 h. The cytotoxic effects of LPS on differentiated PC12 cells were examined with the 3-[4,5-dimethylthiazol-2-yl]-2,5 diphenyl tetrazolium bromide (MTT) assay. Briefly, 10 μL of 5 mg/mL MTT was added to the medium and incubated for 4 h at 37°C. After removal of the culture medium, the insoluble purple formazan crystals were dissolved in 100 μL DMSO to result in a violet solution. The absorbance was measured at 570 nm with a microplate reader (Tecan Infinite M 200 PRO, Männedorf, Switzerland). To determine the apoptotic concentration, the inhibitory concentration (IC_50_) was then calculated. Untreated cells were used as the negative control. Cell viability was expressed as a percentage in comparison to the control group. The results were analyzed based on three independent experiments.

### Acridine Orange and Propidium Iodide (AO/PI) Staining

Double-fluorescent dye acridine orange and propidium iodide staining method was used to analyze the cell viability and the morphological changes occur during apoptosis of differentiated PC12 cells with LPS at pre-conditioning concentrations (3, 6, 9, and 12 μg/mL) for 12 h and post-incubated with 0.75 mg/mL LPS for 12, 24, 48, and 72 h. The treated cells were harvested and washed twice using phosphate-buffered saline (PBS) to remove the remaining media. The cell pellets were resuspended in PBS, followed by the addition of 10 μg/mL of each dye. Stained cells were observed with an Inverted Research Microscope (ECLIPSE Ti-S, Nikon) at 10× magnification within 30 min before the fluorescence signal started to fade. The criteria for identification were as follows: (a) green intact nucleus: viable cells, (b) dense green areas of chromatin condensation in the nucleus: early apoptosis, (c) dense orange areas of chromatin condensation: late apoptosis, and (d) orange intact nucleus: secondary necrosis. The results were analyzed based on three independent experiments.

### Cell Cycle Analysis

To monitor the cell cycle phase, differentiated PC12 cells were seeded in 6-well plates at 2 × 10^5^ cells/well and treated with LPS at pre-conditioning concentrations (3, 6, 9, and 12 μg/mL) for 12 h and post-incubated with 0.75 mg/mL LPS for 12, 24, 48, and 72 h. After each incubation period, cells were detached and washed with PBS three times by spinning down the cells. The resulting pellet was fixed with 80% ethanol and stored at −20°C for one week. Fixed cells were washed with PBS, followed by the addition of 50 μg/mL RNAse and staining with 50 μg/mL propidium iodide (PI) (Sigma-Aldrich, USA). PI binds to DNA as well as RNA. Thus, the addition of RNAse was essential to allow PI to bind to RNA directly to obtain an accurate cell-cycle profile. The cells were incubated for 30 min under dark conditions. Sample acquisition was performed using a flow cytometer (Becton Dickinson, USA). The cell distributions in phases of SubG_0_/G_1_, G_0_/G_1_, S, and G_2_/M were analyzed using a FACSCalibur flow cytometer (Becton Dickinson, USA) from three independent experiments.

### Phosphatidylserine Externalization Analysis

Annexin V-FITC (fluorescein isothiocyanate)/PI dual staining was used to determine the number of cells undergoing apoptosis within a population based on a previously published protocol using the Annexin-V-FITC Apoptosis Detection Kit (BD Pharmingen, USA). Differentiated PC12 cells were seeded in 6-well plates at 2 × 10^5^ cells/well and incubated overnight to allow attachment. Then, the LPS-induced cells and LPS-pre-conditioned cells were stained with 5 μL FITC-conjugated Annexin-V and 5 μL PI according to the manufacturer's instructions. The stained cells were resuspended and incubated for 15 min at room temperature in the dark before being subjected to flow cytometry analysis using a FACSCalibur flow cytometer. The results were analyzed based on three independent experiments.

### Image Analysis for NF-κB Translocation

The NF-κB translocation in LPS-induced and LPS-pre-conditioned cells were determined by using the NF-κB activation kit according to manufacturer's instructions (Cellomics, Thermo Scientific, USA). Briefly, the LPS-induced and LPS-pre-conditioned cells were fixed with formalin, permeabilized with 0.1% Triton X-100 in TBS for 10 min at room temperature, and blocked with 1% Blocker BSA for 15 min at room temperature. Cells were probed with NF-κB/p65 polyclonal antibody at a dilution of 1:50 for at least 1 h at room temperature, washed with PBS, and incubated with DyLight 488 goat anti-rabbit IgG secondary antibody at a dilution of 1:400 for 30 min at room temperature. Hoechst dye was used to stain the nuclei (blue). Fluorescent images were observed and captured at 20× magnification using Inverted Research Microscope (ECLIPSE TI-S, Nikon). The differences between the fluorescence intensity of nuclear and cytoplasmic NF-κB were quantified from three independent experiments.

### Measurement of Cytokines and Chemokines Production

Cytokine and chemokine production was measured in LPS-induced and LPS-pre-conditioned cell culture supernatant using the Proteome Profiler Rat Cytokine Array Kit (R&D system, USA). The extracted proteins from LPS-induced and LPS-pre-conditioned cells were mixed with a cocktail of biotinylated detection antibodies. The sample/antibody mixture was incubated with the Rat Cytokine Array Panel A membrane. The cytokine/detection antibody complex presented with its cognate immobilized antibody that was captured on the membrane. This was followed by a wash to remove unbound material Streptavidin-HRP. Next, chemiluminescent detection reagents were applied. The cytokine array was quantified by scanning the membrane on a Biospectrum AC ChemiHR 40 (UVP, Upland, CA). The array images collected from two independent replicates were analyzed using an image analysis software according to manufacturer's instruction.

### Measurement of Reactive Oxygen Species (ROS) Production

The LPS-induced and LPS-pre-conditioned cells were incubated with 10 mM DCFH-DA for 30 min at 37°C, then washed with PBS twice. The production of ROS was measured using 2′,7′-dichlorofluorescein diacetate (DCFH-DA) which passively enters the cells and reacts with ROS to form the highly fluorescent compound dichlorofluorescein (DCF). The relative levels of fluorescence were quantified using fluorescence microplate reader (Tecan Infinite M 200 PRO, Mannedorf, Switzerland) with excitation at 485 nm and emission at 520 nm. Three independent experiments were performed.

### Measurement of Nitric Oxide (NO) Production

Accumulation of nitrite in the medium was determined by Griess assay. The LPS-induced and LPS-pre-conditioned cells were added in media without phenol red. A total of 100 μL of culture supernatant was reacted with an equal volume of Griess reagent (2.5% phosphoric acid [Merck, Darmstadt, Germany], 1% sulfanilamide [Sigma, St. Louis, Missouri, USA], 0.1% *N*-(1-napthyl)ethylenediamine dihydrochloride [Sigma, St. Louis, Missouri, USA]) in 96-well cell culture plates for 10 min at room temperature in the dark. Nitrite concentrations were determined using standard solutions of sodium nitrite prepared in cell culture medium. The absorbance was determined using a microplate reader (Tecan Infinite M 200 PRO, Mannedorf, Switzerland) at 530 nm. Each assay was repeated in three independent experiments.

### Caspase Assay

To investigate the role of caspases in LPS-pre-conditioned, LPS-induced apoptosis, and LPS-induced apoptosis with caspase inhibitors. Caspase-3/7, caspase-8, and caspase-9 activities were measured based on a published protocol using the Caspase-Glo® assay kit (Promega, Madison, USA). The differentiated PC12 cells were seeded at 1 × 10^5^ cells/well in 96-well plates and allowed to attach overnight. The cells were then induced with (i) 0.75 mg/mL LPS for 24 h, (ii) pre-treated with 3 μg/mL LPS for 12 h and post-incubated with 0.75 mg/mL LPS for 12 h, and (iii) pre-treated with Z-VAD-FMK, a pan-caspase inhibitor and Z-DEVD-FMK, a selective caspase-3 inhibitor for 3 h and post-incubated with 0.75 mg/mL LPS. At the end of the treatment period, 100 μL of Caspase-Glo-3/7, Caspase-Glo-8^*^, and Caspase-Glo-9 reagents were added to each well. The plates were gently shaken using a plate shaker, and the luminescence signal was measured after 30 min using a microplate reader (Infinite M200 Pro Tecan, Austria). The caspase activities were expressed as a fold of the untreated control treatment. Three independent experiments were performed.

### Western Blotting of Cytoplasmic Proteins and Nuclear Protein

The differentiated PC12 cells were induced with (i) 0.75 mg/mL LPS for 24 h, (ii) pre-treated with 3 μg/mL LPS for 12 h and post-incubated with 0.75 mg/mL LPS for 12 h and (iii) pre-treated with Z-VAD-FMK, a pan-caspase inhibitor and Z-DEVD-FMK, a selective caspase-three inhibitor for 3 h and post-incubated with 0.75 mg/mL LPS. After treatment, the total proteins of cells were extracted by using Universal Protein Extraction Reagent (Bioteke Corporation) containing 20 mM Tris (pH 7.5), sodium chloride (NaCl), ethylenediaminetetraacetic acid (EDTA), sodium pyrophosphate, special non-ionic detergents, complete phosphatase inhibitors, and 1% protease inhibitor cocktail. The protein concentrations were quantified by BCA protein assay kit (Bioteke Corporation). Briefly, a standard plot was generated by using bovine serum albumin (BSA) and equivalent amounts (25 μg) of each sample were subjected to 12% sodium dodecyl sulfate-polyacrylamide gel electrophoresis (SDS-PAGE) to separate the proteins based on their molecular weight and were transferred onto PVDF membranes. The membranes were incubated with 5% non-fat dry milk (Amersham ECL Advance blocking agent) in TBST for 90 min. Then, the membranes were incubated with primary antibodies (anti-TNF-α, Bax, Bcl-2, anti-caspase-3, p53, c-MYC, and Hsp70 proteins) (Santa Cruz Biotechnology, INC, CA, US) in 1:1000 dilution at 4°C overnight, followed by incubation with secondary antibodies for 2 h at room temperature. The immunoblots were visualized by enhanced chemiluminescence procedure using Clarity™ Western ECL Substrate western blotting kit (Bio Rad, USA). The densitometry of the gel bands, including beta actin loading control, was analyzed using ImageJ (NIH). Each immunoblot was repeated in three independent experiments.

### Extraction of Nuclear NF-κB for Western Blot

The differentiated PC12 cells were induced with (i) 0.75 mg/mL LPS for 24 h, (ii) pre-treated with 3 μg/mL LPS for 12 h and post-incubated with 0.75 mg/mL LPS for 12 h, and (iii) pre-treated with Z-VAD-FMK, a pan-caspase inhibitor and Z-DEVD-FMK, a selective caspase-three inhibitor for 3 h and post-incubated with 0.75 mg/mL LPS. After treatment, the nuclear and cytosolic protein of the cells were extracted as previously described. The supernatants containing cytosolic proteins were removed and stored at −80°C and the nuclear pellet was resuspended in cold PBS (20 mM Tris (pH 7.5), sodium chloride (NaCl), ethylenediaminetetraacetic acid (EDTA), sodium pyrophosphate, special non-ionic detergents, and phosphatase inhibitors (Universal Protein Extraction Reagent, Bioteke Corporation) containing complete 1% protease inhibitor cocktail on ice. After centrifugation at 13,000 rpm for 20 min at 4°C, supernatants containing nuclear proteins were removed and stored at −80°C (Abdi et al., 2011).

### ELISA

Commercially available ELISA assays (United States Biological, Massachusett, US) were used to measure the expressions of p53, c-MYC, and Hsp70 proteins. Cell culture supernatants were collected from the differentiated PC12 cells that had been stimulated with (i) 0.75 mg/mL LPS for 24 h, (ii) pre-treated with 3 μg/mL LPS for 12 h and post-incubated with 0.75 mg/mL LPS for 12 h, and (iii) pre-treated with Z-VAD-FMK, a pan-caspase inhibitor and Z-DEVD-FMK, a selective caspase-3 inhibitor for 3 h and post-incubated with 0.75 mg/mL LPS. Induced cells were added to three different 96-well microplates. The plates were pre-coated with monoclonal anti-Hsp70, anti-c-MYC, and anti-p53 antibodies for 1 h. After the incubation period, the wells were decanted and washed five times. Then, the wells were incubated with a substrate for HRP enzyme. Finally, stop solution was added to terminate the reaction. The intensity of color was measured spectrophotometrically at 450 ± 10 nm using a microplate reader (Infinite M200 Pro Tecan, Austria). The protein concentrations in each sample were interpolated from the standard curve. The assay was performed in triplicate.

### RNA Extraction, cDNA Synthesis, and Real-Time PCR

Total ribonucleic acid (RNA) was extracted using RNeasy Plus Mini Kit (Qiagen, Germany) from the differentiated PC12 cells induced with (i) 0.75 mg/mL LPS for 24 h, (ii) pre-treated with 3 μg/mL LPS for 12 h and post-incubated with 0.75 mg/mL LPS for 12 h, and (iii) pre-treated with Z-VAD-FMK, a pan-caspase inhibitor and Z-DEVD-FMK, a selective caspase-3 inhibitor for 3 h and post-incubated with 0.75 mg/mL LPS. Briefly, cells were dislodged from the bottom of the flask using a cell scraper and transferred into a 15 mL centrifuge tube. Cells were centrifuged at 1,800 g for 5 min. Then, the cells were lysed with a lysis solution that disrupted cell membranes and was capable of protecting the RNA from endogenous RNases. Subsequently, the cells were homogenized by pipetting vigorously and vortexing. The homogenate was then mixed with ethanol thoroughly and centrifuged at 10,000 *g* for 30 s through a microfilter cartridge supplied with silica-based membrane that selectively binds RNA. The impurities were effectively removed by a specific washing step. Finally, total RNA was eluted by running the elution solution through an elution cartridge. Homogenates were kept on ice to prevent RNase activity. Purified RNA was used for reverse transcription. The concentration and purity of RNA were measured using NanoDrop™ (Thermo Scientific, USA). Then, cDNA was synthesized by RT^2^ First Strand Kit (Qiagen, Germany) and followed by adding the cDNA to RT^2^ SYBR Green Mastermix (Qiagen, Germany). These PCR component mixes were dispensed into a 96-well plate RT^2^ Profiler PCR Arrays (Qiagen, Germany) which contained 84 wells of disease-focused genes, five wells of housekeeping genes, one well of genomic DNA control, three wells of reverse-transcription controls, and three wells of positive PCR controls. The plates were placed in a real-time cycler (Biosystem StepOne Plus, Thermo Scientific, USA). The data were analyzed by using SABiosciences PCR Array Data Analysis (www.SABiosciences.com/pcrarraydataanalysis.php). All samples were processed in triplicate.

### Data Analysis and Statistics

All data were obtained based on two or three independent experiments and expressed as mean ± SEM. The results were analyzed by Graphpad Prism 6 using two-way ANOVA followed by Bonferroni *post-hoc* comparison test. The symbols ^*^p < 0.05, ^**^p < 0.01, ^***^p < 0.001, and ^****^p < 0.0001 indicate a significant difference. Western blot results were evaluated using computer-based ImageJ software, and the densities were calculated in arbitrary units.

## Results

### The Morphology of PC12 Cells

**Figure 1:**
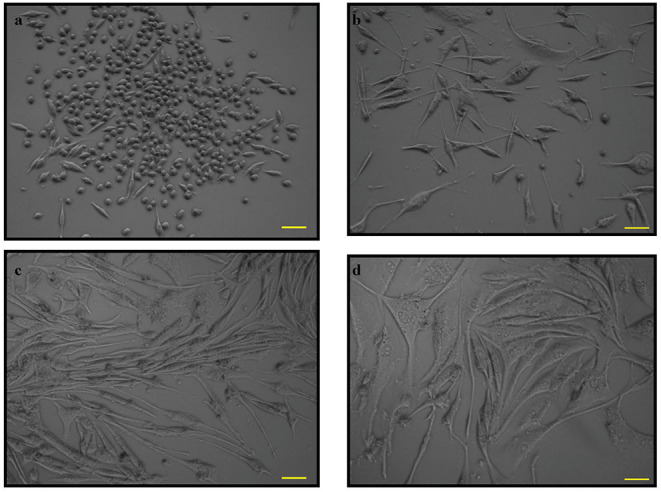
The phase contrast microscopy images of PC12 cells. PC12 cells with absence of nerve growth factor (NGF) at **(a)**. 12 h and **(b)**. 24 h and PC12 cells with NGF at **(c)**. 48 h and **(d)**. 72 h. Pictomicrographs were taken with original magnification (10×); the scale bar represents 100 μm.

The morphology of PC12 cell lines was observed from cells grown in a T25 flask (Figure 1). The cell body was distinct from neighboring cell bodies. Figures 1c,d show PC12 cells induced by nerve growth factor (NGF) to differentiate into neuron-like cells with elongated neurites. However, specific parameters for the morphology were not measured.

### Effect of LPS on Viability of Differentiated PC12 Cells

**Figure 2:**
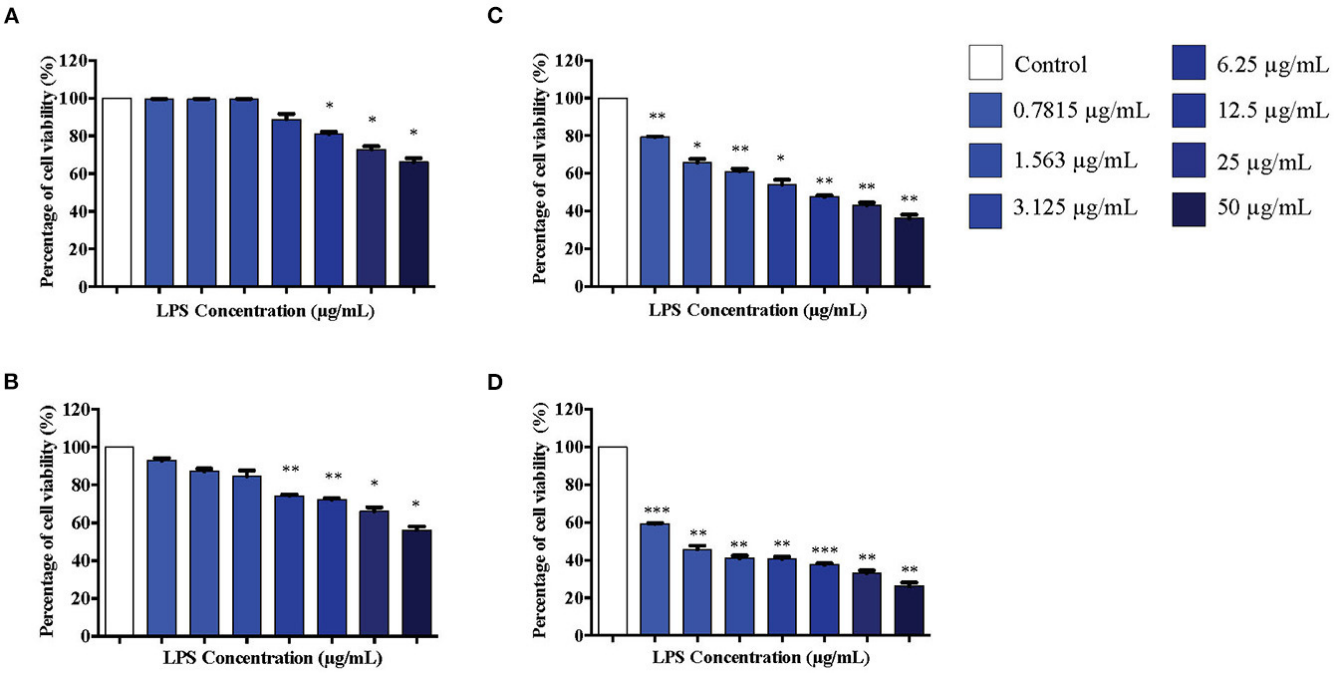
The differentiated PC12 cells were exposed to 50, 25, 12.5, 6.25, 3.125, 1.563, and 0.7815 μg/mL LPS for **(A)**. 12, **(B)**. 24, **(C)**. 48, and **(D)**. 72 h. Cell viability was determined by MTT assay. Bars represent mean data ± S.E.M from three independent experiments. Data were analyzed using one-way ANOVA, followed by a Bonferroni *post-hoc* test. Percentage of cell viability of LPS-stimulated cells was compared to control. *p < 0.05, **p < 0.01, ***p < 0.001 considered to be statistically significant.

Differentiated PC12 cells were treated with serial concentrations of LPS for 12 to 72 h and MTT assay was conducted to determine the cell viability. No significant cell death was observed in cells treated with 0.7815, 1.563, 3.125, and 6.25 μg/mL LPS at 12 h (Figure 2A). At 24 h, cells were still viable in response to 0.7815, 1.563, and 3.125 μg/mL LPS where the treatment did not affect 85% of the cell viability, but there was cell death at this time point in response to 6.25 μg/mL LPS onwards (Figure 2B). However, at 48 and 72 h, cell viability decreased significantly in a concentration-dependent manner (Figures 2C,D respectively).

### Effect of LPS Pre-conditioning on Differentiated PC12 Cells

**Figure 3:**
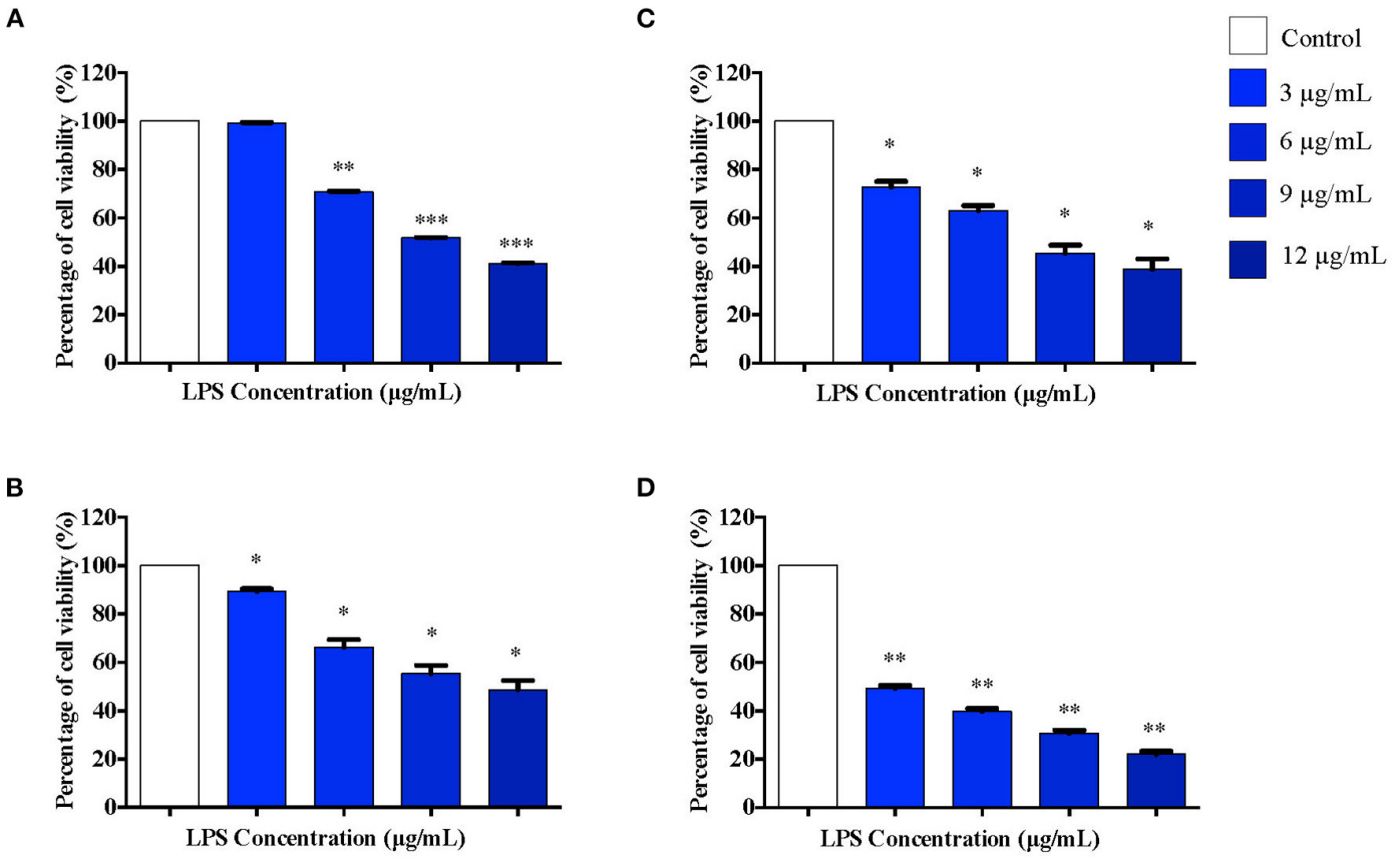
The differentiated PC12 cells were exposed to 3, 6, 9, and 12 μg/mL LPS for **(A)**. 12, **(B)**. 24, **(C)**. 48, and **(D)**. 72 h. Cell viability was determined by MTT assay. Bars represent mean data ± S.E.M from three independent experiments. Data were analyzed using one-way ANOVA, followed by a Bonferroni *post-hoc* test. Percentage of cell viability of LPS-stimulated cells was compared to control. *p < 0.05, **p < 0.01, ***p < 0.001 considered to be statistically significant.

Based on our observations from MTT assay, we performed further studies by stimulating differentiated PC12 cells with 3, 6, 9, and 12 μg/mL LPS for 12 h to obtain the appropriate pre-conditioning concentration for the cells. Following that, the cells were exposed to a higher concentration of LPS, 0.75 mg/mL, which has been shown to induce apoptosis in these cells. Cell viability was measured using MTT assay following 12, 24, 48, and 72 h of LPS stimulation. Interestingly, no significant toxicity was observed for the cells treated with 3 μg/mL LPS for 12 h compared to the control cells (Figure 3A). Although cell viability was not affected in response to 3 μg/mL LPS at 12 h, cell viability decreased significantly at 6 μg/mL LPS onwards at 12 h (Figure 3A). For the cells treated with 3, 6, 9, and 12 μg/mL LPS at 24 (Figure 3B), 48 (Figure 3C), and 72 h (Figure 3D), the cell viability was not maintained and decreased significantly in a concentration-dependent manner.

### Effect of LPS Pre-Conditioning on Cell Viability Observed From Acridine Orange and Propidium Iodide (AO/PI) Staining

**Figure 4:**
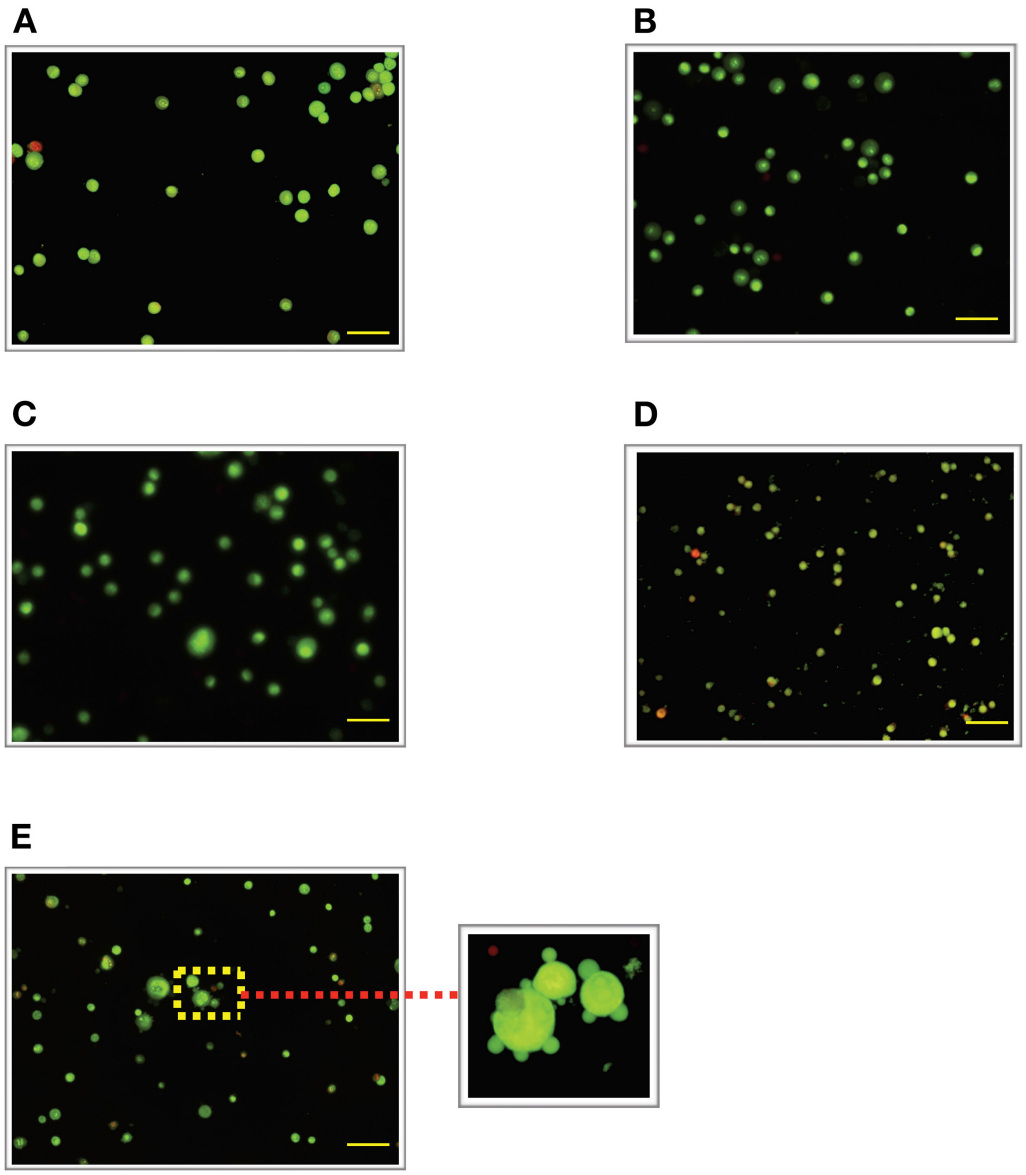
Acridine orange and propidium iodide (AO/PI) nucleic acid binding dyes in LPS-stimulated cells. Cells were stimulated with 3 μg/mL LPS for **(A)**. Control, **(B)**. 12, **(C)**. 24, **(D)**. 48, and **(E)**. 72 h. Cells were stained with AO/PI to examine their morphological changes, where the green color represents AO, which stains live and dead nucleated cells while the red color represents PI, which stains dead nucleated cells. Representative fluorescence images from inverted fluorescence microscope. Field of views for each time point from three independent experiments. Purple arrow shows plasma membrane blebbing. Pictomicrographs were taken with original magnification (10×); the scale bar represents 100 μm.

**Figure 5:**
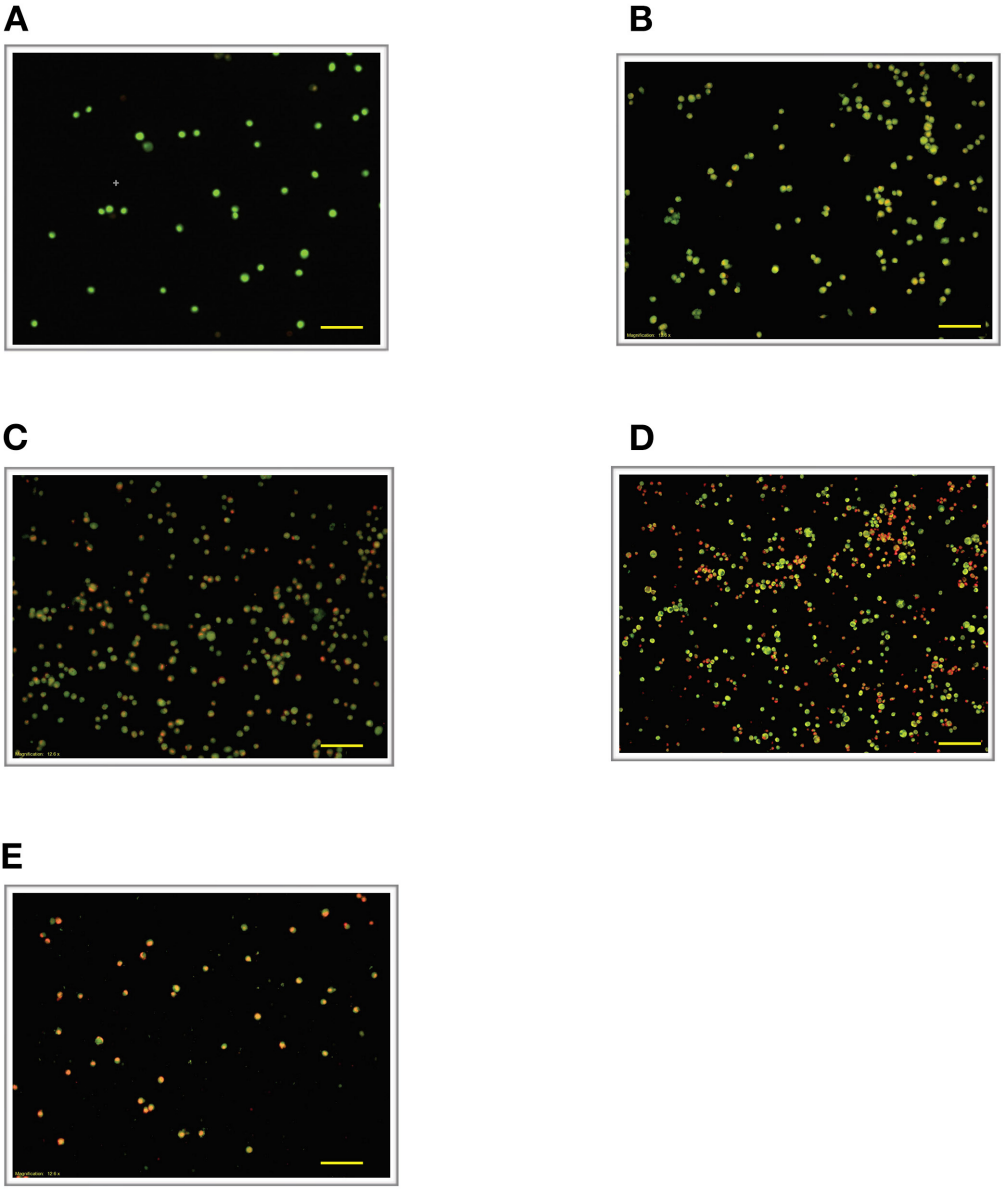
Acridine orange and propidium iodide nucleic acid binding dyes in LPS-stimulated cells. Cells were stimulated with 6 μg/mL LPS for **(A)**. Control, **(B)**. 12, **(C)**. 24, **(D)**. 48, and **(E)**. 72 h. Cells were stained with AO/PI to examine their morphological changes, where the green color represents AO, which stains live and dead nucleated cells while the red color represents PI, which stains dead nucleated cells. Representative fluorescence images from inverted fluorescence microscope. Field of views for each time point from three independent experiments. The cells lost membrane integrity with yellow to orange color change, indicating pre-early and early apoptosis. Pictomicrographs were taken with original magnification (10×); the scale bar represents 100 μm.

**Figure 6:**
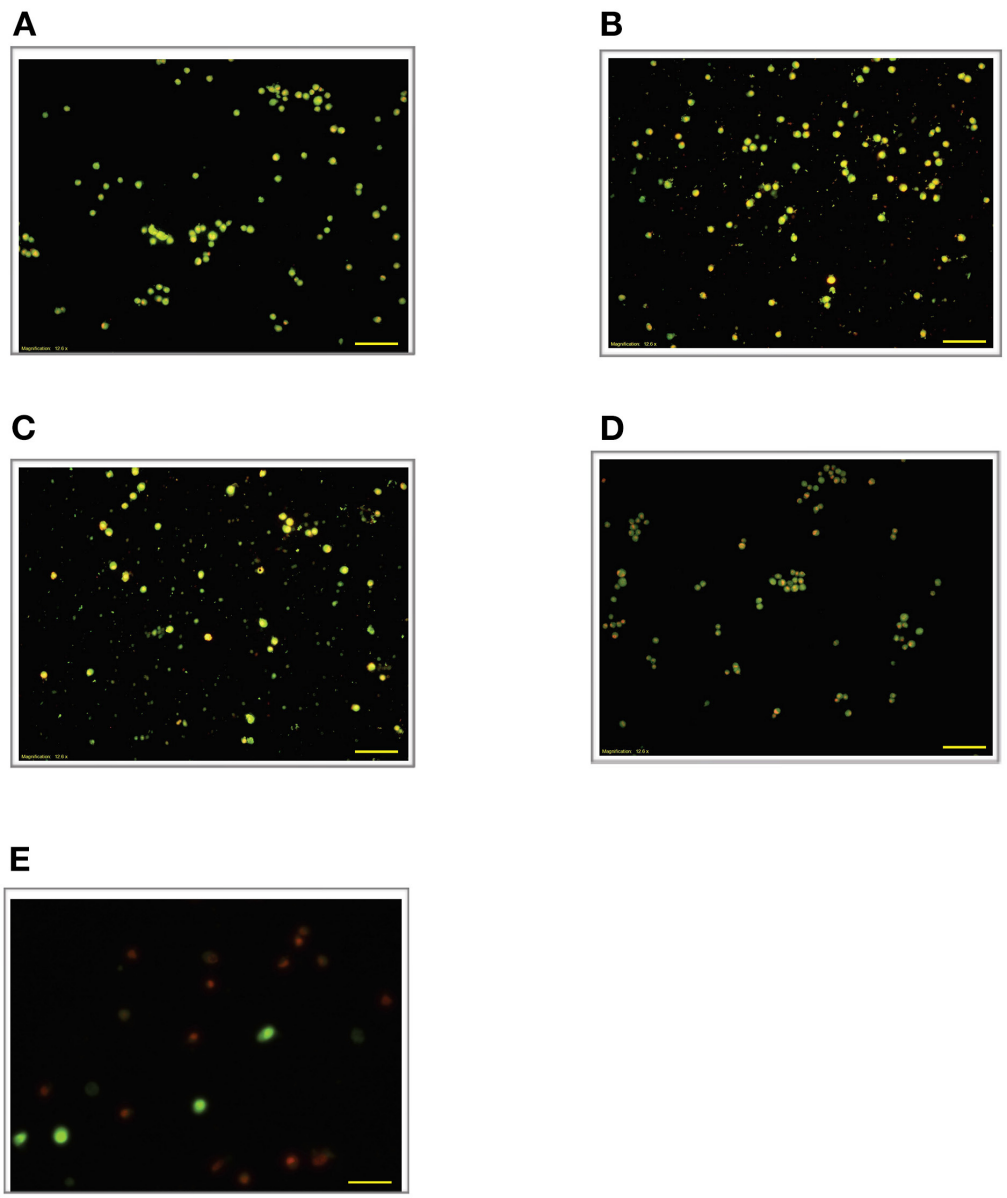
Acridine orange and propidium iodide nucleic acid binding dyes in LPS-stimulated cells. Cells were stimulated with 9 μg/mL LPS for **(A)**. Control, **(B)**. 12, **(C)**. 24, **(D)**. 48, and **(E)**. 72 h. Cells were stained with AO/PI to examine their morphological changes, where the green color represents AO, which stains live and dead nucleated cells while the red color represents PI, which stains dead nucleated cells. Representative fluorescence images from inverted fluorescence microscope. Field of views for each time point from three independent experiments. The cells lost membrane integrity with yellow to orange color change, indicating pre-early and early apoptosis, and orange-reddish, indicating late apoptosis. Pictomicrographs were taken with original magnification (10×); the scale bar represents 100 μm.

**Figure 7:**
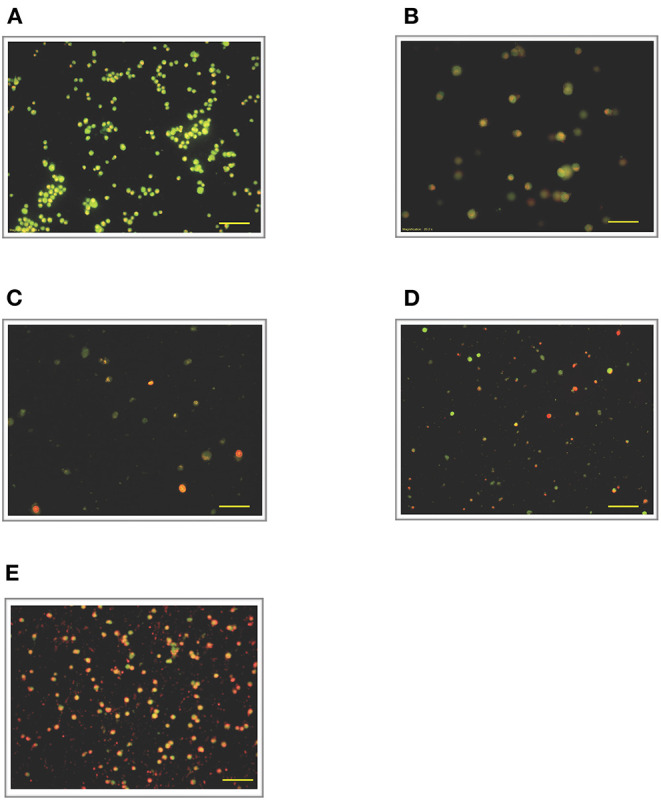
Acridine orange and propidium iodide nucleic acid binding dyes in LPS-stimulated cells. Cells were stimulated with 12 μg/mL LPS for **(A)**. Control, **(B)**. 12, **(C)**. 24, **(D)**. 48, and **(E)**. 72 h. Cells were stained with AO/PI to examine their morphological changes, where the green color represents AO, which stains live and dead nucleated cells while the red color represents PI, which stains dead nucleated cells. Representative fluorescence images from inverted fluorescence microscope. Field of views for each time point from three independent experiments. The cells lost membrane integrity with orange-reddish color, indicating late apoptosis; red cells indicate necrosis. Pictomicrographs were taken with original magnification (10×); the scale bar represents 100 μm.

To validate our findings, we examined the morphological changes of LPS-pre-conditioned cells using AO/PI staining method. Pre-conditioned cells with LPS 3 μg/mL for 12 h followed by post incubation with 0.75 mg/mL LPS prominently exhibited intact green nuclear structure and had well-preserved cellular morphology as control cells (Figure 4B). Perhaps, the cells treated with 3 μg/mL LPS after 24 h showed pre-early apoptosis (Figures 4C,D). However, the cells treated with 3 μg/mL LPS after 72 h showed blebbing of the plasma membrane (Figure 4E). Notably, the cells pre-treated with other concentrations of LPS (6, 9, and 12 μg/mL) for 12 h followed by post-incubation with 0.75 mg/mL LPS for another 12 h showed typical morphologies of apoptotic cells such as blebbing; condensation of chromatin; loss of membrane integrity; yellow-, orange-, and red-colored cells in a time-dependent manner, which indicate early (after 24 h) and late apoptosis (48 and 72 h) (Figures 5–7). The orange-reddish cells indicate late apoptosis and necrosis which occurred at 72 h (Figure 7). The AO/PI double staining method confirmed that LPS pre-conditioning with 3 μg/mL LPS for 12 h protected the cells against the apoptotic concentration of LPS.

### Cytoprotective Effect of LPS Pre-Conditioning at G_0_/G_1_ Phase in Differentiated PC12 Cells

**Figure 8:**
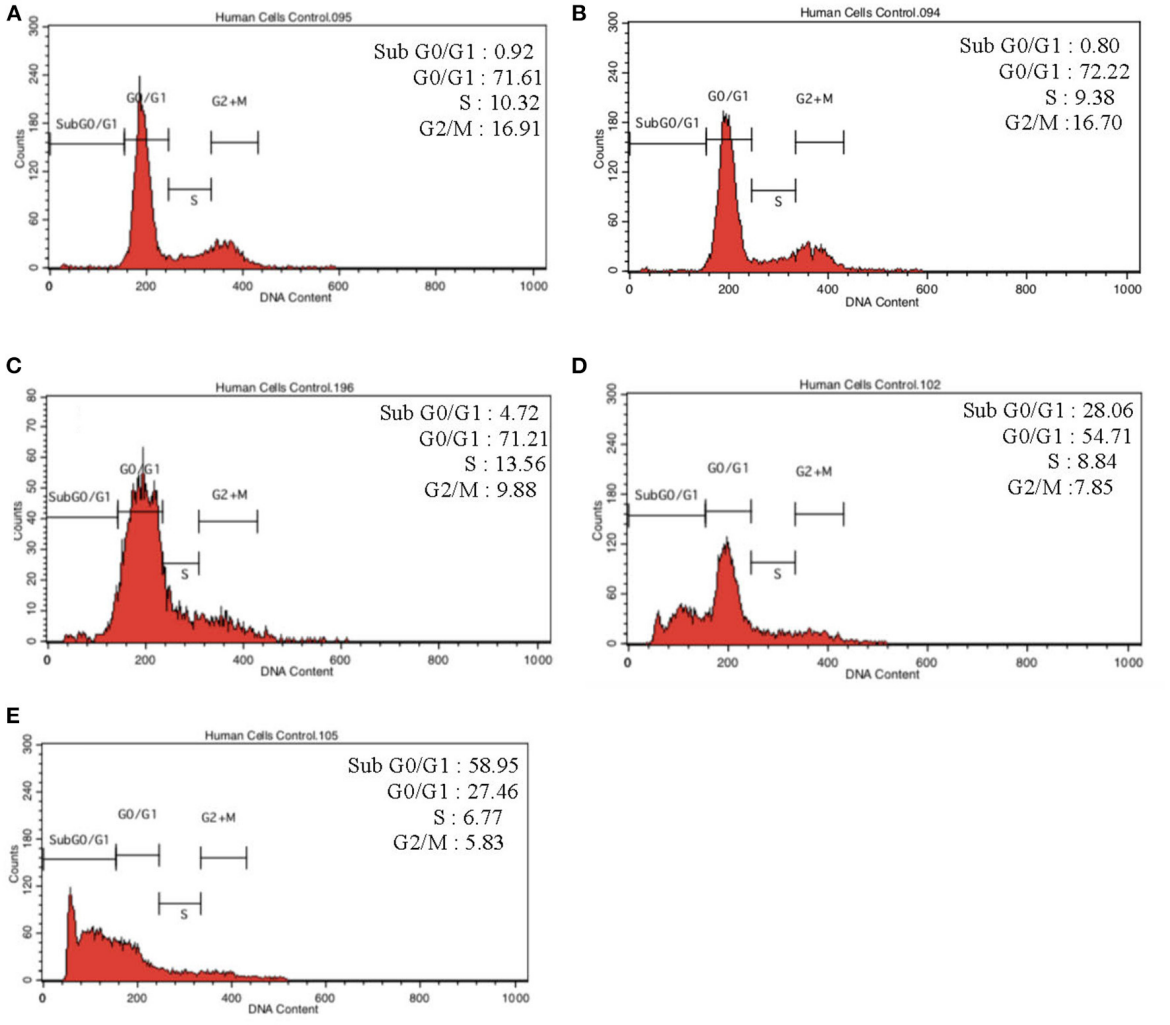
Cell cycle distribution analysis in LPS-preconditioned cells by using flow cytometry. Cells were treated with 3 μg/mL LPS for **(A)**. Control, **(B)**. 12, **(C)**. 24, **(D)**. 48, and **(E)**. 72 h. Representative flow cytometry patterns from three independent experiments are shown.

**Figure 9:**
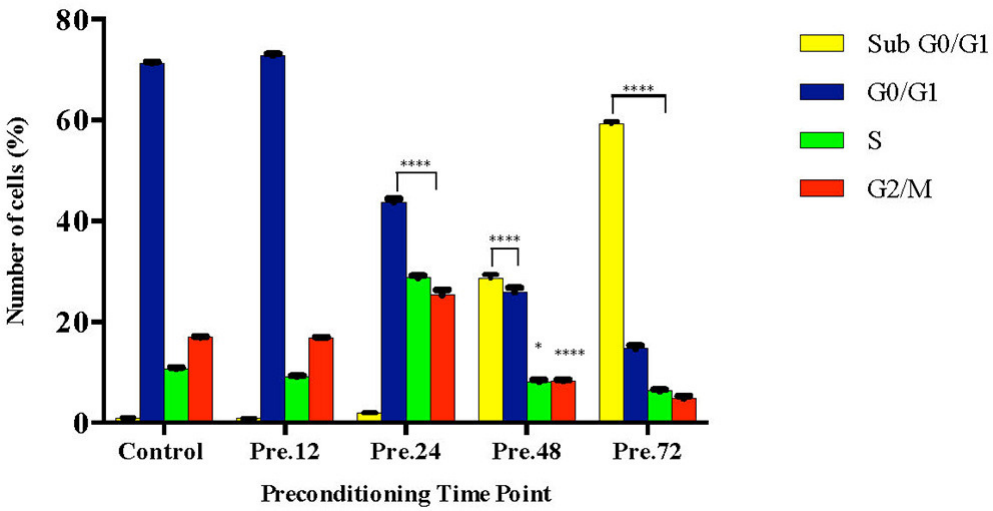
Quantitative analysis of cell cycle distribution analysis in LPS-preconditioned cells by using flow cytometry. The differentiated PC12 cells were exposed to 3 μg/mL LPS for 12, 24, 48, and 72 h. Cell cycle phase distribution based on the DNA content was determined by cell cycle assay. Bars represent mean data ± S.E.M from three independent experiments. Data were analyzed using one-way ANOVA, followed by a Bonferroni *post-hoc* test. Percentage of LPS-stimulated cells was compared to the control. *p < 0.05, ****p < 0.0001 considered to be statistically significant.

**Figure 10:**
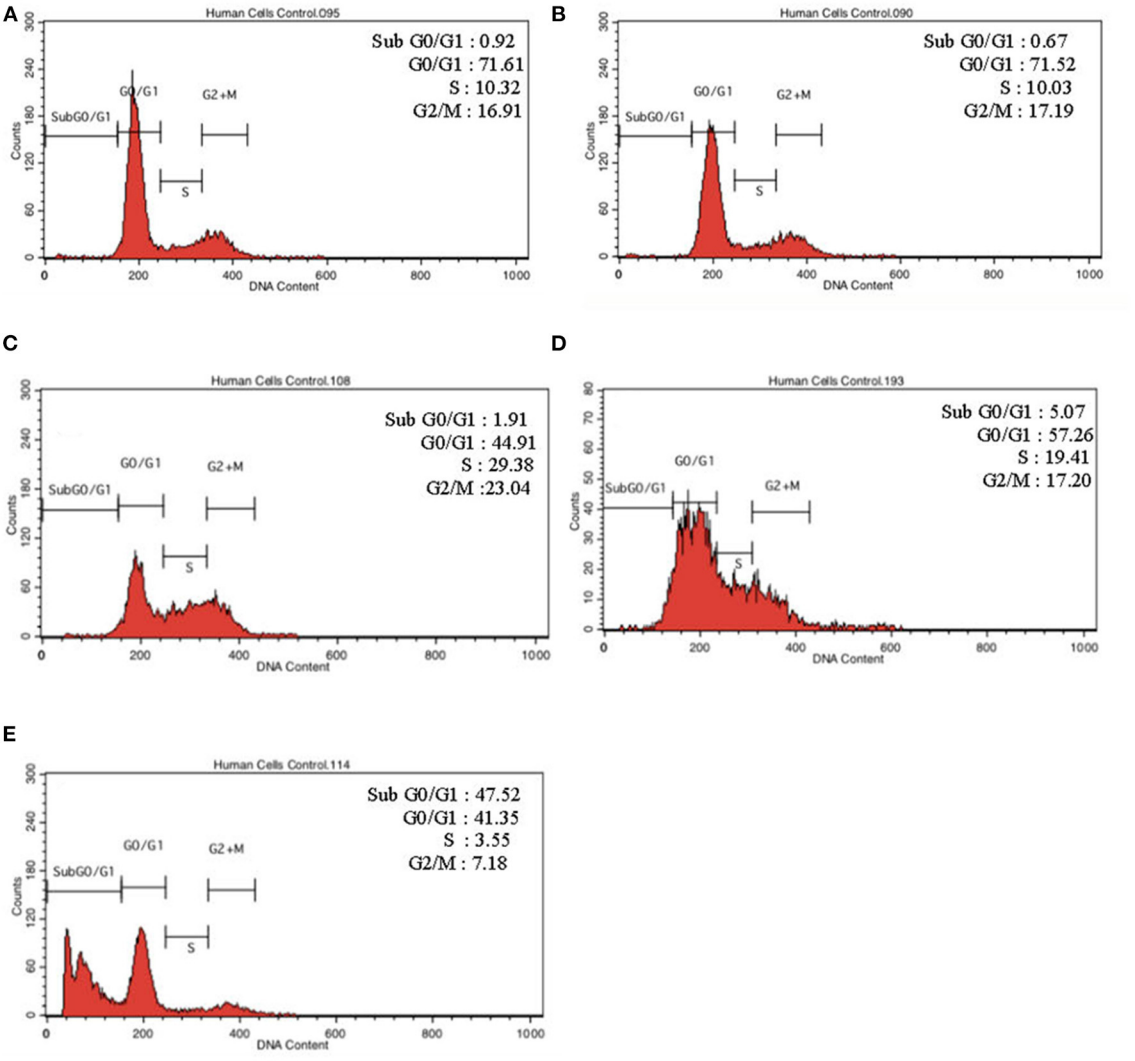
Cell cycle distribution analysis in LPS-preconditioned cells by using flow cytometry. Cells were treated with 6 μg/mL LPS for **(A)**. Control, **(B)**. 12, **(C)**. 24, **(D)**. 48, and **(E)**. 72 h. Representative flow cytometry patterns from three independent experiments are shown.

**Figure 11:**
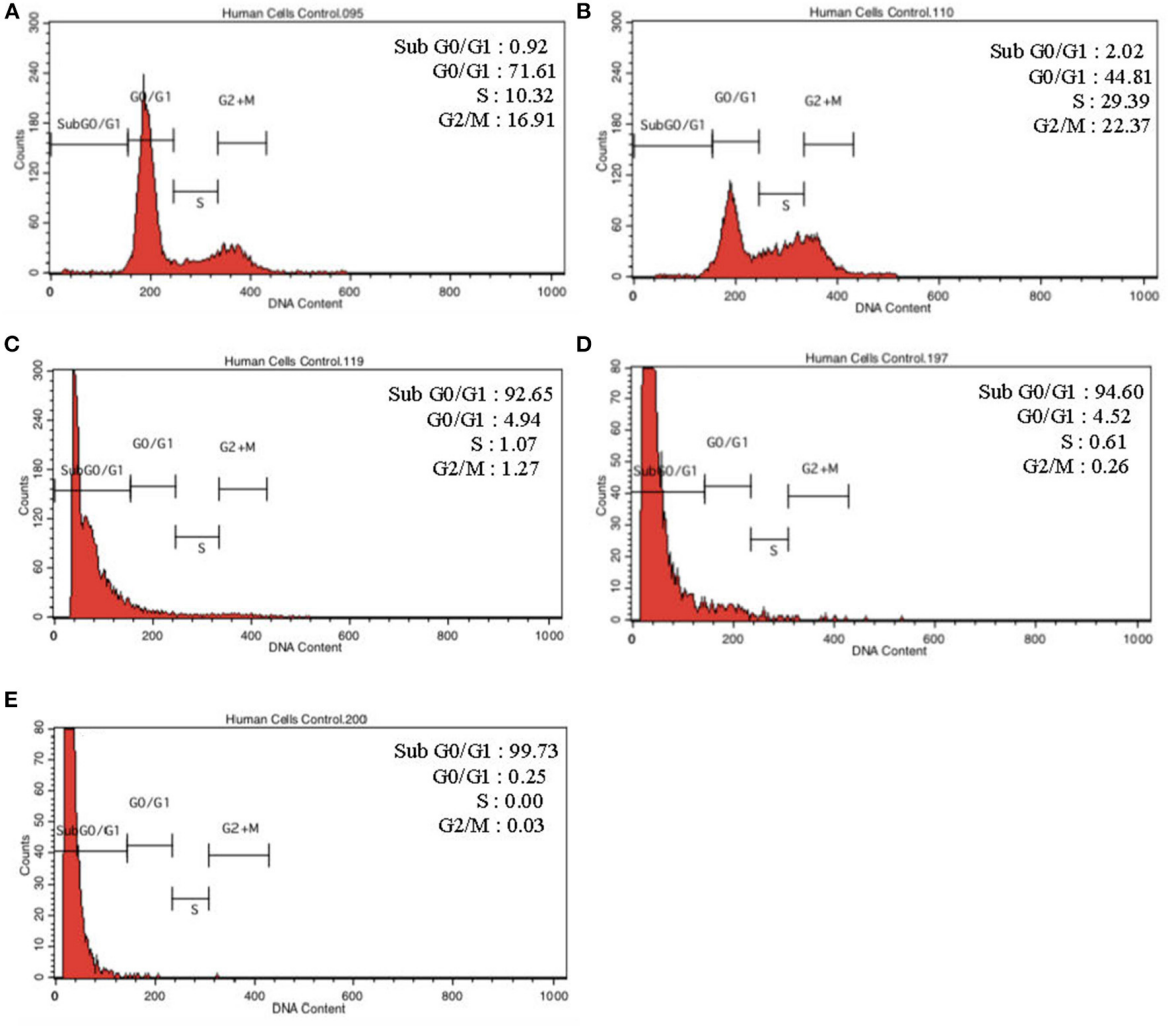
Cell cycle distribution analysis in LPS-preconditioned cells by using flow cytometry. Cells were treated with 9 μg/mL LPS for **(A)**. Control, **(B)**. 12, **(C)**. 24, **(D)**. 48, and **(E)**. 72 h. Representative flow cytometry patterns from three independent experiments are shown.

To further validate our findings, we narrowed down the LPS pre-conditioning concentration to investigate cell cycle progression. Differentiated PC12 cells pre-treated with 3, 6, and 9 μg/mL LPS for 12 h and subsequently exposed to 0.75 mg/mL LPS for another 12, 24, 48, and 72 h were analyzed via flow cytometry. From the result, the number of cells treated with 3 μg/ml LPS at 12 h exhibited similar patterns as control cells (Figure 8B). Nonetheless, the number of cells stimulated with 3 μg/ml LPS at 24, 48, and 72 h (Figures 8C–E) decreased in a time-dependent manner compared to the control cells as shown in Figure 9. Yet, the number of cells treated with 6 and 9 μg/mL showed an increase in subG_0_/G_1_ and a decrease in G_0_/G_1_, S, and G_2_/M in a time-dependent manner (Figures 10, 11). In line with our findings from MTT assay and AO/PI double staining, we chose pre-exposure to 3 μg/mL of LPS for 12 h as the protective condition for the cells.

### LPS Pre-conditioning Inhibits Cell Apoptosis

**Figure 12:**
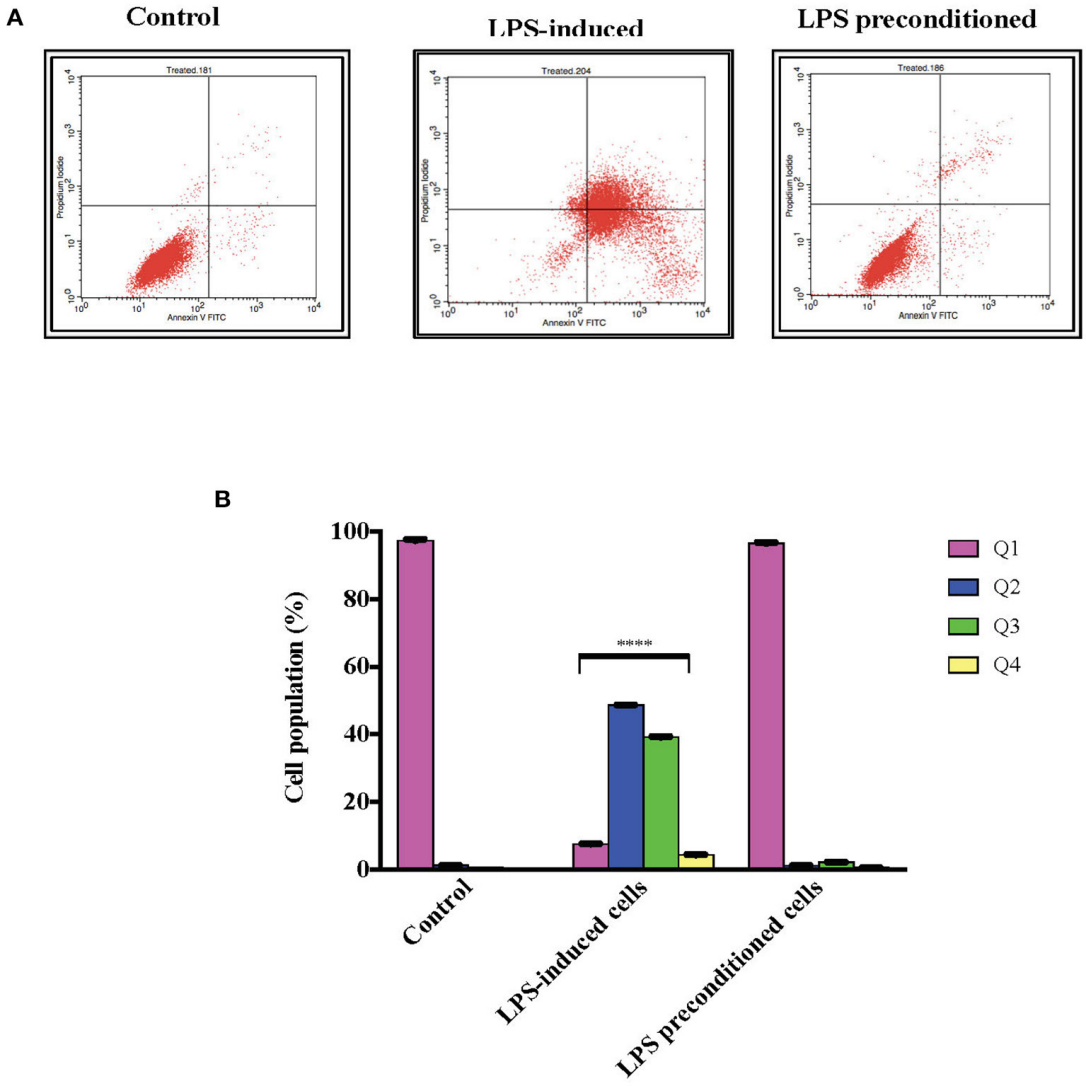
Quantitative analysis of apoptosis progression within population in LPS-preconditioned cells by using flow cytometry. Annexin V-FITC and PI staining of the LPS-induced and LPS-preconditioned cells at the indicated time point earlier measured by flow cytometry. **(A)**. There is a distinct and gradual cell population shift from the lower left quadrant to the lower right, upper right, and upper left, indicating a gradual increment of cells with exposed phosphatidylserine. Q1 is the Annexin V-FITC–/PI– quadrant (intact cells), Q2 is the Annexin V-FITC+/PI– quadrant (early apoptotic cells), Q3 is the Annexin V-FITC+/PI+ quadrant (late apoptotic cells), and Q4 is the Annexin V-FITC–/PI– quadrant (dead cells). Bars represent mean data ± S.E.M from three independent experiments. **(B)**. Data were analyzed using one-way ANOVA, followed by a Bonferroni *post-hoc* test. Percentage of LPS-stimulated cells was compared to the control. ****p < 0.0001 considered to be statistically significant.

Annexin V-FITC staining is a standard procedure to monitor the progression of apoptosis. The LPS-induced and LPS-pre-conditioned cells were stained with Annexin V-FITC, in which early apoptotic cells showed Annexin V+ and PI– whereas late (end-stage) apoptotic cells will demonstrate Annexin V+/PI+. As shown in Figure 12A, the pre-conditioned cell population are found in the lower left quadrant as control cells. However, the population of LPS-induced cells shifted from the left lower quadrant to the lower right quadrant followed by the upper right quadrant. It is obvious that the LPS-pre-conditioned cells are protected from cell death against the apoptotic concentration; this result is consistent with our findings from MTT assay, AO/PI double staining, and cell cycle study. This further justifies that 3 μg/mL LPS for 12 h is the most appropriate pre-conditioning concentration that can offer cytoprotective effect in differentiated PC12 cells and hence, this was used as pre-conditioning concentration for subsequent experiments in this study.

### LPS-Induced NF-κB Nuclear Translocation

**Figure 13:**
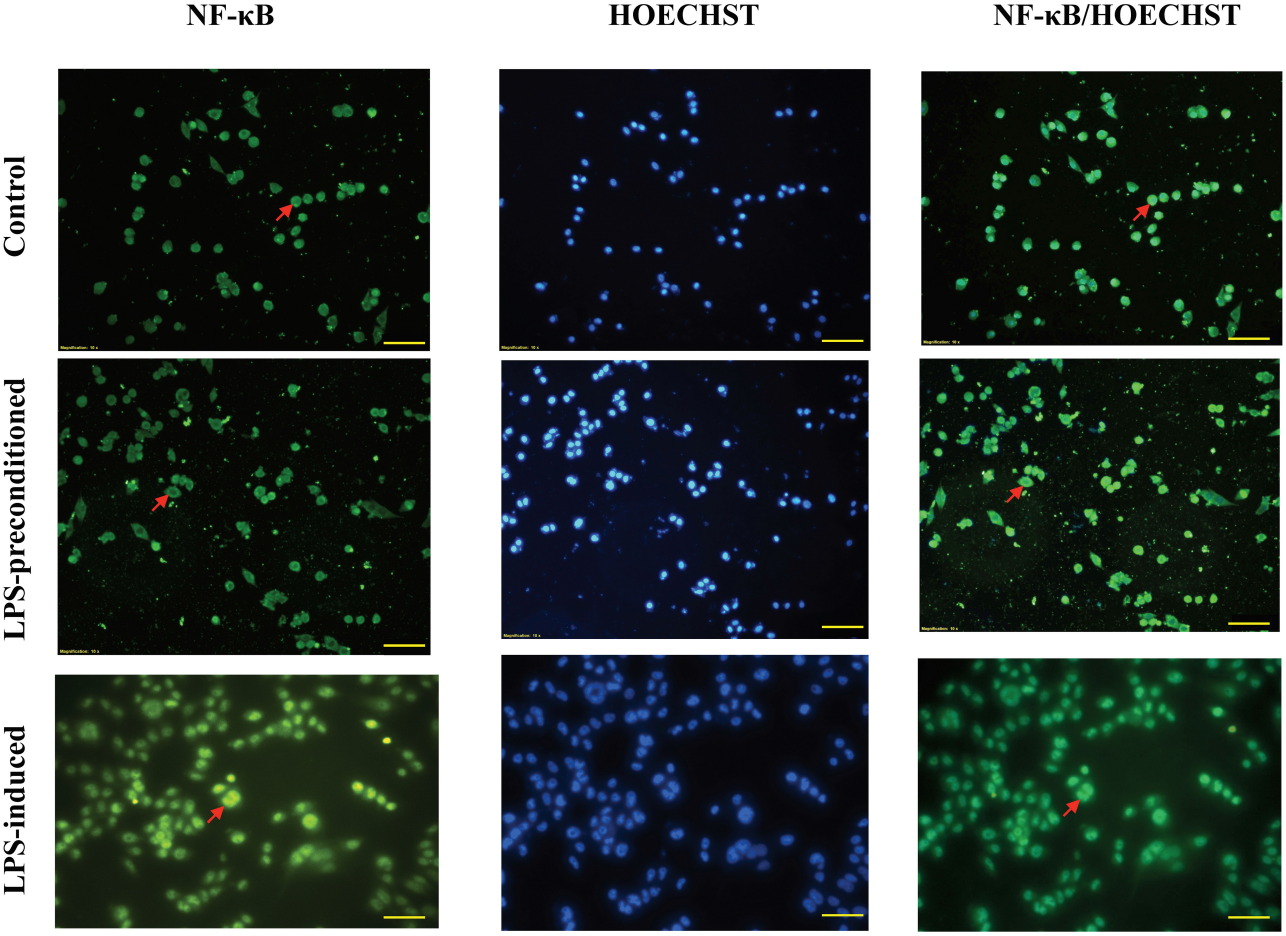
Immunofluorescence analysis of NF-κB activation. The nuclear translocation was determined by the blue fluorescence (Hoechst staining) and the NF-κB/p65 subunit by its green fluorescence. Representative fluorescence images from inverted fluorescence microscope. Field of views for each time point from three independent experiments. The translocation of NF-κB/p65 from the cytoplasm into the nucleus indicated the activation of the transcription factor. Pictomicrographs were taken with original magnification (20×); the scale bar represents 100 μm.

NF-κB is a transcription factor which has a role in inflammation, neuronal survival, differentiation, apoptosis, neuron outgrowth, and synaptic plasticity. To observe the translocation of NF-κB, an immunofluorescence assay was conducted which was correlated with the secretion of inflammatory cytokines. As shown in (Figure 13), in LPS-induced cells, NF-κB fluorescence intensity in the cytoplasm was absent and instead was predominantly observed in the nucleus, which indicates the translocation of NF-κB from the cytoplasm into the nucleus. However, the LPS-pre-conditioned cells exhibited higher NF-κB fluorescence signal in the cytoplasm and lower in the nucleus, which indicates no translocation of NF-κB from the cytoplasm into the nucleus. In the control cells, NF-κB fluorescent signal was intensified in the cytoplasm but absent in the nucleus, indicating that NF-κB translocation did not occur in these cells.

### LPS Pre-conditioning Suppresses the Expression of Pro-inflammatory Cytokines and Chemokines

**Figure 14:**
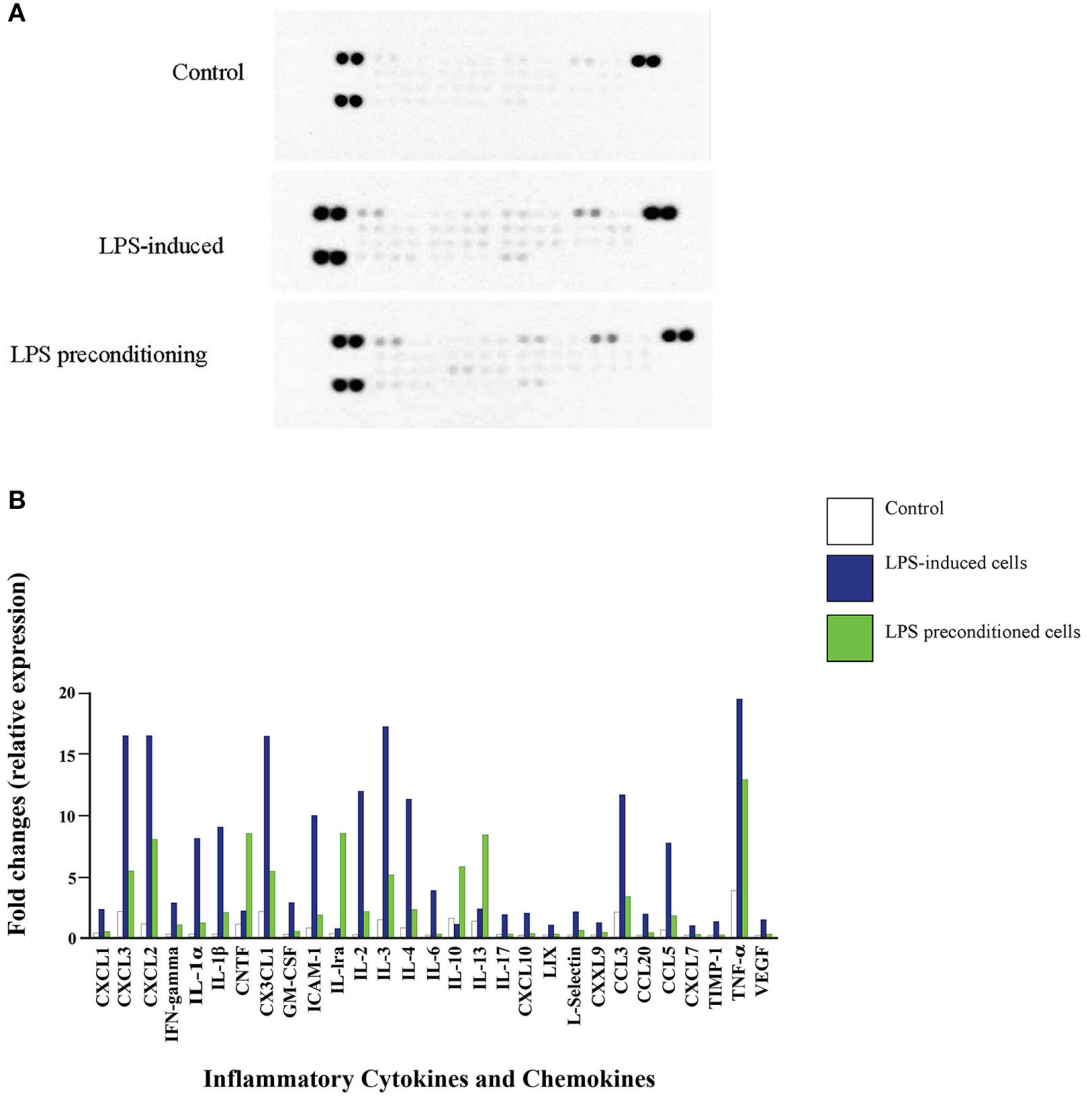
Quantitative analysis of inflammatory cytokines and chemokines. **(A)** The Rat Cytokine Array detects multiple analytes in LPS- induced cells, LPS-preconditioned cells, and control cells. Spot densities were analyzed by scanning the membrane on a Biospectrum AC ChemiHR 40 densitometer, and data were normalized to control and expressed as fold change relative to the control. **(B)** Quantitative analysis in the arrays showed differences in cytokine and chemokine expression in LPS-induced cells, LPS-preconditioned cells, and control cells. Each experiment was performed in duplicate.

We investigated whether pre-exposure to LPS would have an impact on the expression of pro- and anti-inflammatory cytokines, which are critical in the immune and inflammatory response of the neuronal cells. Equal amount of 300 μg of protein from the LPS-induced and LPS-pre-conditioned cells were lysed and the inflammatory cytokines were screened using Proteome Profiler Rat Cytokine Array Kit (R & D system, Inc.). Interestingly, pro-inflammatory cytokines interleukin-1 alpha (IL-1α), interleukin-1 beta (IL-1β), interleukin-2 (IL-2), interleukin-3 (IL-3), interleukin-4 (IL-4), interleukin-6 (IL-6), interleukin-17 (IL-17), interferon-gamma (IFN-γ), and tumor necrosis factor-alpha (TNF-α) were highly expressed in LPS-induced cells compared to LPS-pre-conditioned cells (Figure 14). However, anti-inflammatory cytokines interleukin-10 (IL-10), interleukin-13 (IL-13), and interleukin-1 receptor antagonist (IL-1Ra) and chemokine ciliary neurotrophic factor (CNTF) were downregulated in LPS-induced cells compared to LPS-pre-conditioned cells. Our results suggest that LPS-pre-conditioned cells repress the activation of pro-inflammatory cytokines which facilitates cytoprotection and attenuates apoptosis.

### LPS Pre-conditioning Inhibits ROS Production

**Figure 15:**
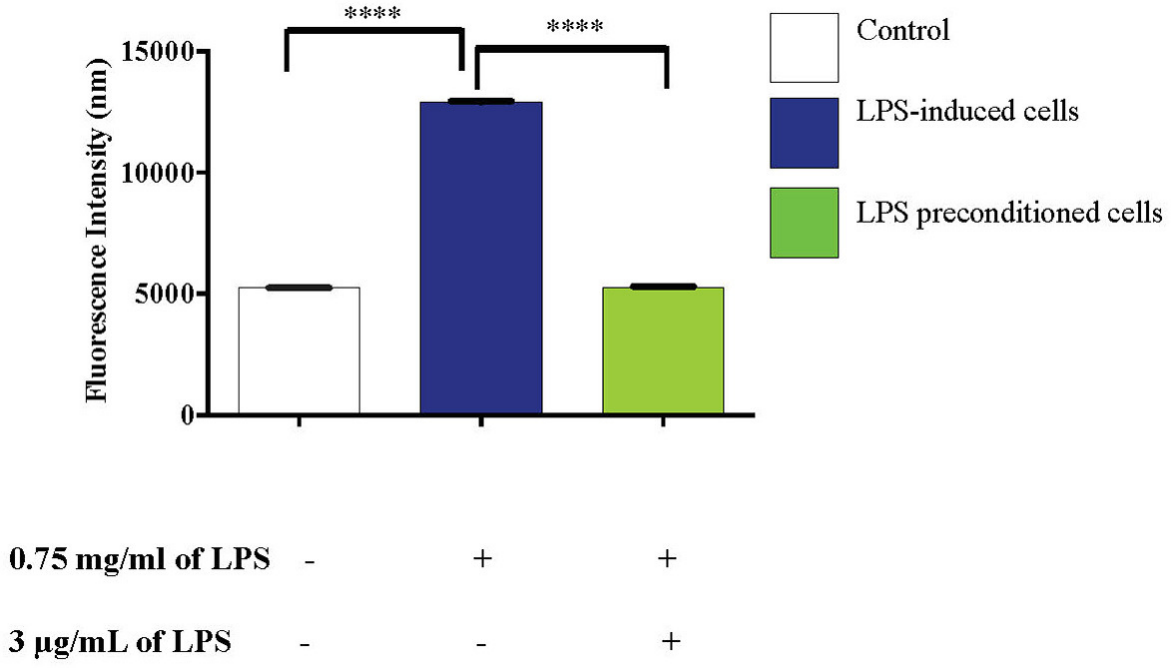
LPS activates pro-inflammatory mediators, ROS. The release of ROS in LPS-induced and LPS-preconditioned cells were measured using DCFH-DA. Bars represent mean data ± S.E.M from three independent experiments. Data were analyzed using one-way ANOVA, followed by a Bonferroni *post-hoc* test. Relative release of fluorescence intensity in LPS-induced cells were compared to the control. ****p < 0.0001 considered to be statistically significant.

The effects of reactive oxygen species (ROS), an important mediator of inflammatory responses and oxidative stress-induced cell death, was studied in LPS-induced and LPS-pre-conditioned cells. The production of oxidative stress was measured using 2′,7′-dichlorofluorescein diacetate (DCFH-DA) and the relative levels of fluorescence were quantified using a fluorescence microplate reader. LPS-induced cells showed a significant increase in the production of ROS (*p* < 0.0001) compared to the LPS-pre-conditioned cells and control cells (Figure 15).

### LPS Pre-conditioning Attenuates NO Production

**Figure 16:**
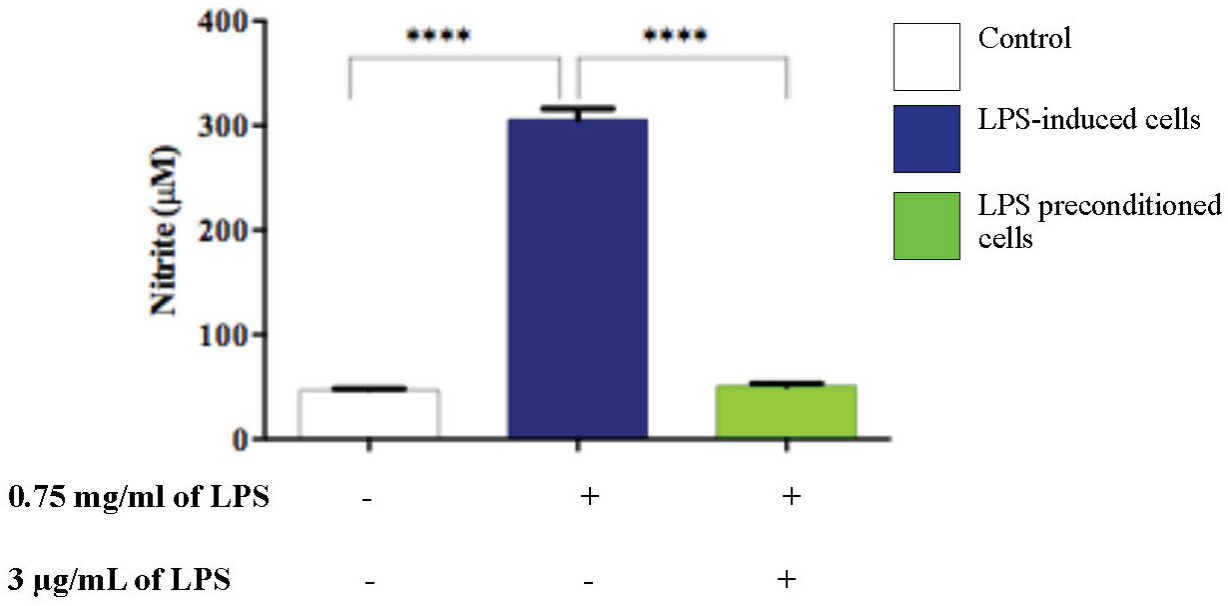
LPS activates pro-inflammatory mediators, NO. The release of NO in LPS-induced and LPS-preconditioned cells were measured using Griess assay. Bars represent mean data ± S.E.M from three independent experiments. Data were analyzed using one-way ANOVA, followed by a Bonferroni *post-hoc* test. Relative release of nitrite in LPS-induced cells were compared to the control. ****p < 0.0001 considered to be statistically significant.

NO is considered to be a pro-inflammatory mediator and excessive release of NO has been shown to be toxic to neurons as it activates neuroinflammation which eventually leads to neurodegeneration. As shown in (Figure 16), the production of NO in the LPS-induced cells was significantly higher (*p* < 0.0001) compared to the control and LPS-pre-conditioned cells. Therefore, these findings indicate that NO synthesis could be prevented through pre-conditioning which confers a protective effect by inhibiting the release of pro-inflammatory mediators.

### LPS Pre-conditioning Downregulates Bax and TNF-α and Upregulates Bcl-2

**Figure 17:**
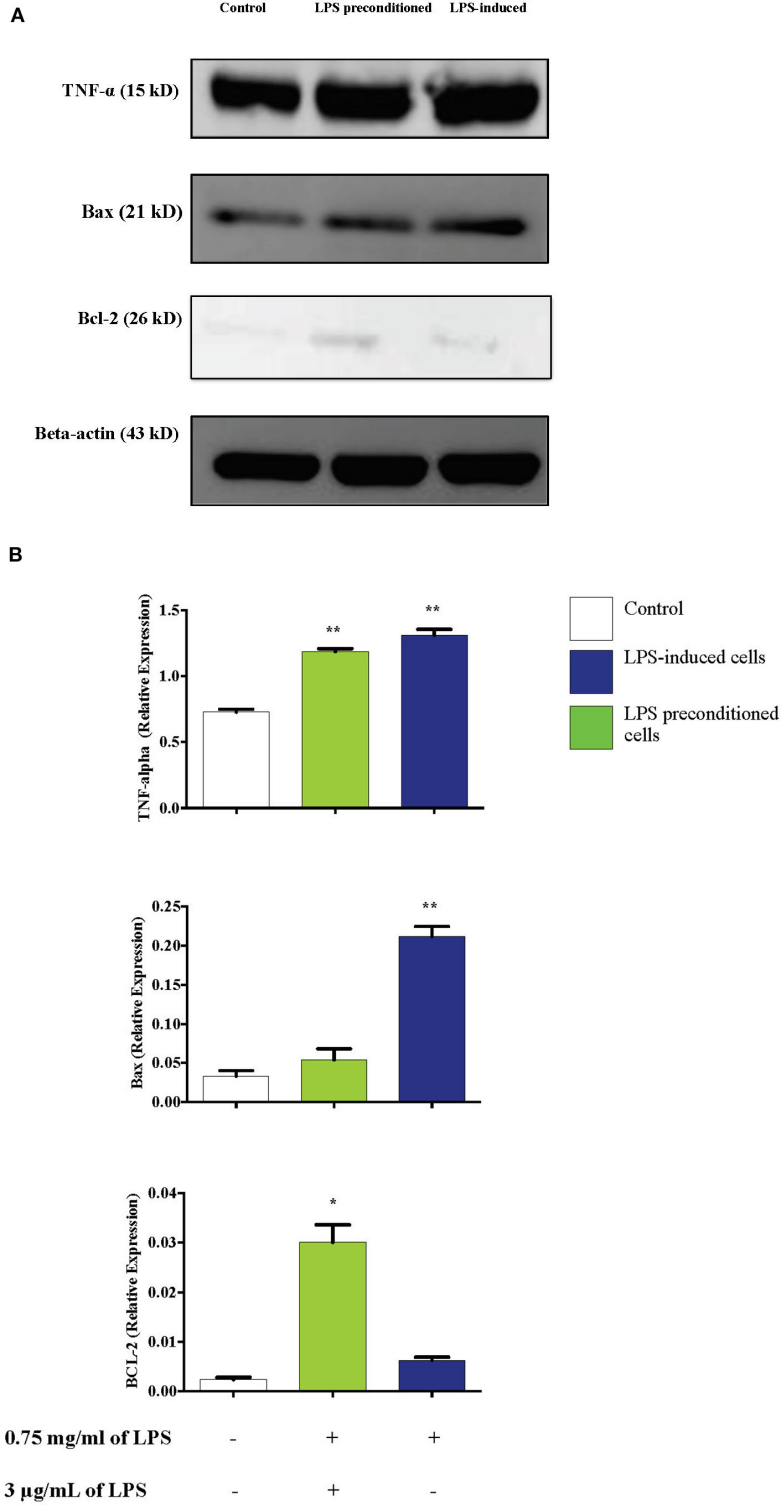
Effect of LPS on TNF-α, Bax, and Bcl-2 protein expressions in LPS-induced and LPS-preconditioned cells. The expressions of inflammatory cytokine, TNF-α; pro-apoptotic protein, Bax; and anti-apoptotic protein, Bcl-2 were determined in whole cell lysates by western blotting. **(A)** Band densities were measured by densitometric analysis, and data were normalized to control and expressed as fold change relative to beta-actin. **(B)** Bars represent data from three independent experiments. TNF-α, Bax, and Bcl-2 expressions in cells that were stimulated with LPS were compared to control. *p < 0.05, **p < 0.01 as determined by one-way ANOVA with Bonferroni's correction.

TNF-α has been documented to have a dual role, mediating apoptosis or cell survival and proliferation. We investigated whether TNF-α modulation of the expression of Bax (pro-apoptotic protein) and Bcl-2 (anti-apoptotic protein) may occur through NF-κB activation, which could modulate apoptosis and cell survival. We used western blotting to detect Bax, Bcl-2, and TNF-α expressions in LPS-induced and LPS-pre-conditioned cells. Expression of both Bax and TNF-α increased in LPS-induced cells compared to the LPS-pre-conditioned cells. Notably, TNF- α expression was also expressed in the LPS-pre-conditioned cells. On the contrary, significant in the expression of Bcl-2 was observed in the LPS-pre-conditioned cells compared to the LPS-induced cells (Figure 17). The previous result confirmed the translocation of NF-κB from the cytoplasm into the nucleus and this could activate the secretion of pro-inflammatory cytokines and pro-apoptotic protein in LPS-induced cells. On the other hand, the inhibition of NF-κB translocation upregulated the expressions of anti-inflammatory cytokines and anti-apoptotic proteins. Therefore, this result confirmed that TNF-α is an inflammatory cytokine that forces cells to undergo programmed cell death which is probably modulated by the upregulation of Bax, leading to enhanced pro-apoptotic effects. However, the inhibition of NF-κB translocation upregulated Bcl-2, leading to cytoprotective effect.

### Caspase Activation

**Figure 18:**
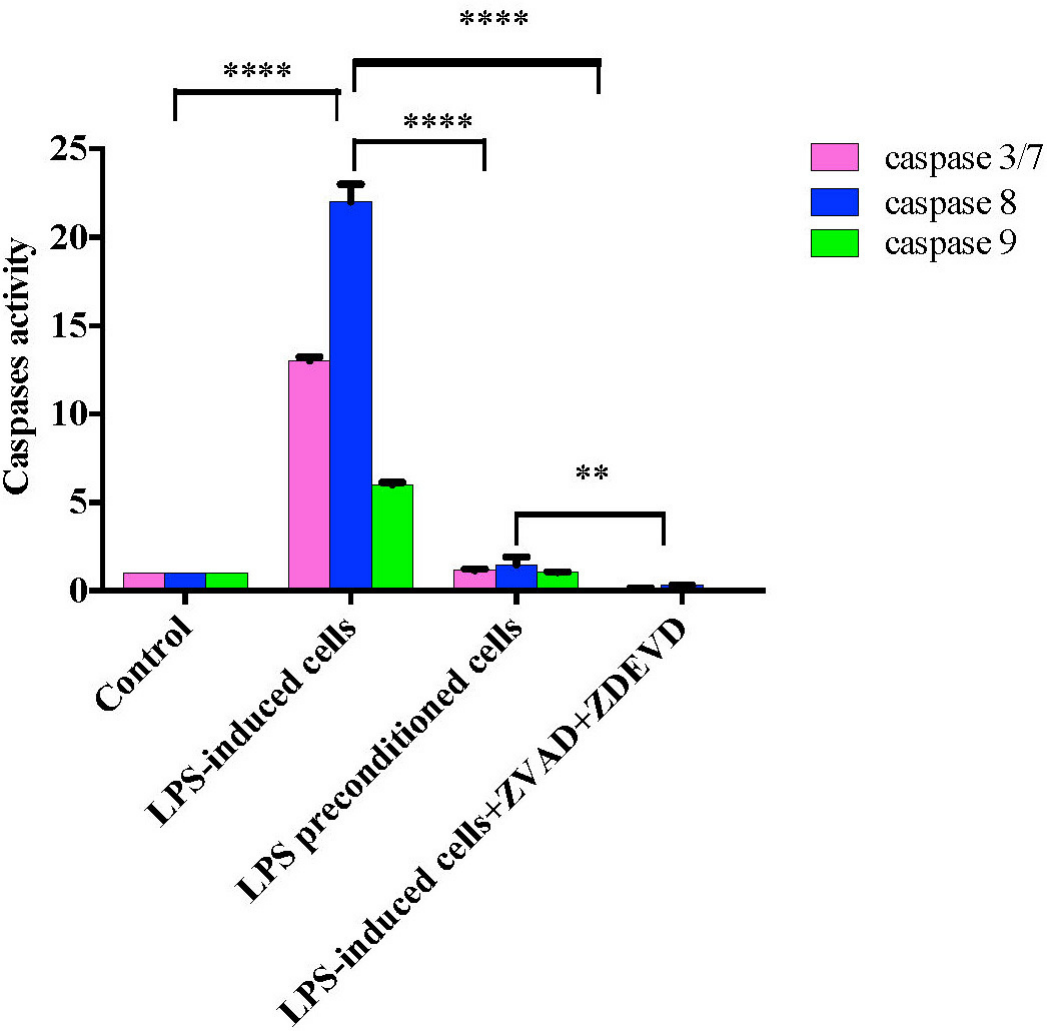
Caspase activity in intrinsic and extrinsic apoptosis pathway. Fold changes in caspase 3/7, caspase-8, and caspase-9 activities in LPS-induced cells, LPS-preconditioned cells, and LPS-induced cells with caspase inhibitors compared to control cells based on the relative luminescence units generated through the cleavage of substrate after treatment. Data were analyzed using two-way ANOVA, followed by a Bonferroni *post-hoc* test, **p < 0.01 ****p < 0.0001 vs. control cells. Data are expressed as mean ± S.E.M of three independent experiments.

Caspases, also known as cysteine aspartic proteases, are the central components of the apoptotic response. The apoptotic caspases are divided into two classes: (i) initiator caspases (caspase 8 and 9) and (ii) effector caspases (caspase 3 and 7). Caspase regulation was investigated by evaluating caspase 3/7, 8, and 9 activities by using Caspase-Glo® assay kit (Promega Corp., USA) in LPS-induced cells, LPS-pre-conditioned cells, and LPS-induced cells in the presence of synthetic non-specific caspase inhibitors. As shown in Figure 18, caspase 8 showed significant increase, followed by caspase 3/7 and caspase 9. The results showed that LPS activates apoptosis pathway via caspase 8 (extrinsic pathway) followed by the activation of caspase 3/7, which is involved in intrinsic pathway. Moreover, the cells treated with caspase inhibitors showed suppression of caspase activity, which resulted in the inhibition of apoptosis pathway.

### LPS Pre-conditioning Represses Caspase-3 Expression

**Figure 19:**
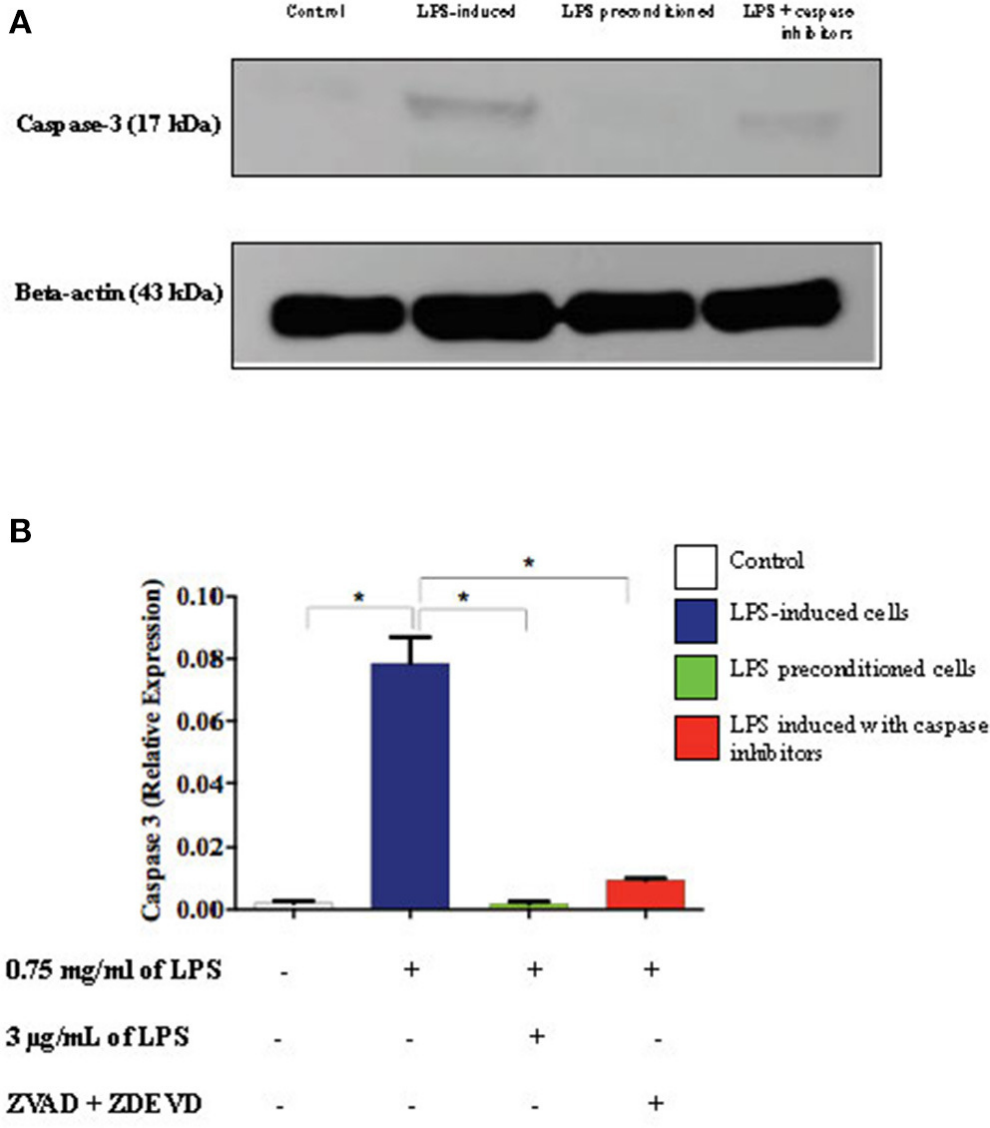
Expressions of caspase-3. **(A)** The expressions of caspase-3 was determined in whole cell lysates by western blotting. Band densities were measured by densitometric analysis, and data were normalized to the control and expressed as fold change relative to beta-actin. **(B)** Bars represent data from three independent experiments. Caspase-3 expressions in LPS-induced cells, LPS-pre-conditioned cells, and LPS-induced cells with caspase inhibitors were compared to the control. *p < 0.05, as determined by one-way ANOVA with Bonferroni's correction.

Caspase-3 is an executioner caspase which is activated in the intrinsic (mitochondrial) and extrinsic (death ligand) apoptotic mechanism. We investigated the expression of caspase-3 in the cell lysates of LPS-induced cells, LPS-pre-conditioned cells, and LPS-induced cells in the presence of synthetic non-specific caspase inhibitors by western blot analysis. LPS-induced cells showed a significant increase in the expression of caspase-3 (*p* < 0.05) compared to the control cells, LPS-pre-conditioned cells, and LPS-induced cells in the presence of a synthetic non-specific caspase inhibitor (Figure 19). This confirmed that caspase-3 expression was successfully inhibited by using a synthetic non-specific caspase inhibitor Z-VAD-FMK (pan-caspase inhibitor) and Z-DEVD-FMK (selective caspase-3 inhibitor).

### LPS Effect on Translocation of Nuclear Factor-kappa B (NF-κB) From Cytosol to Nucleus

**Figure 20:**
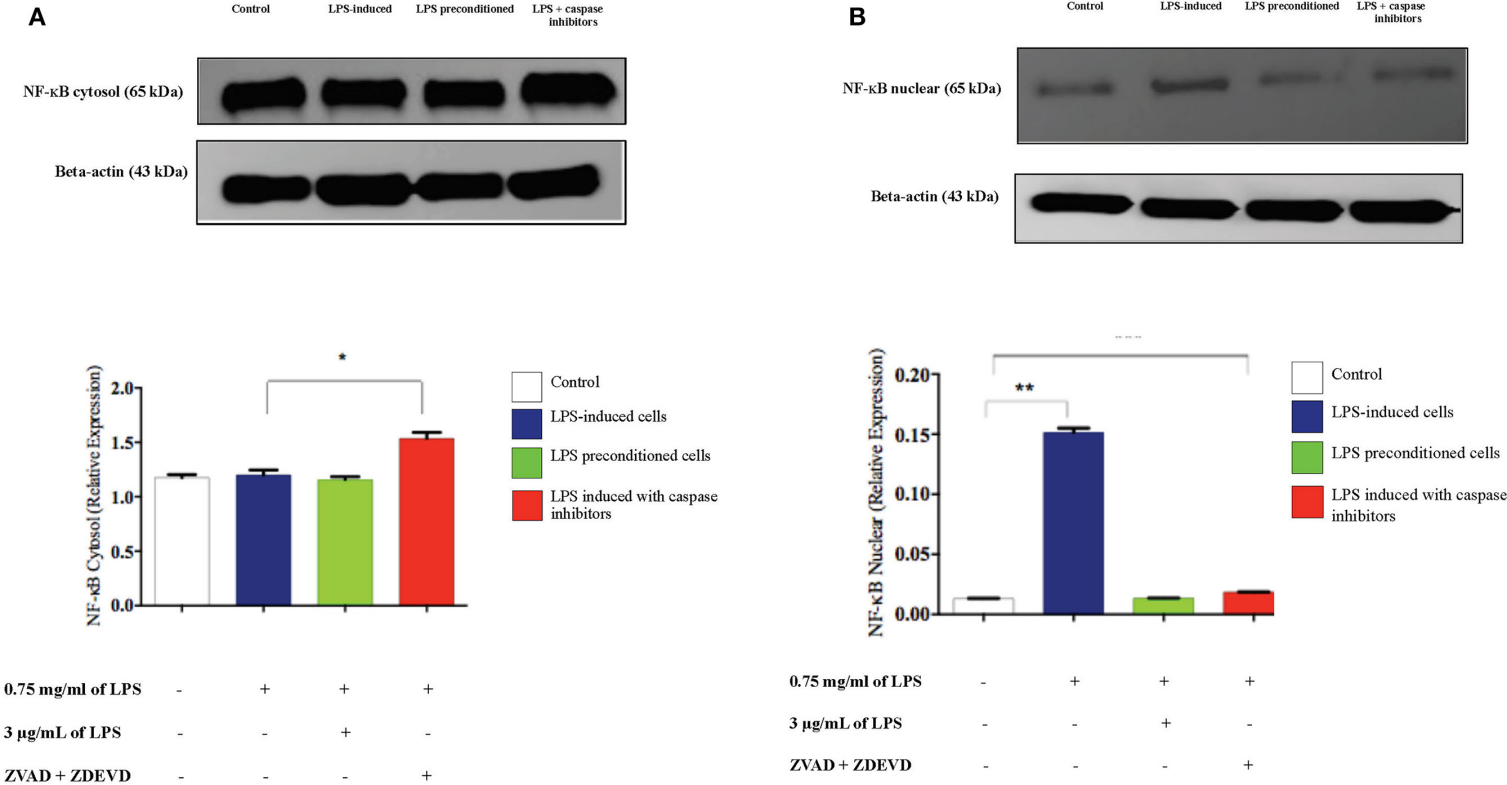
Analysis of cytoplasmic and nuclear expression of NF-κB by western blot. The cytoplasmic **(A)**. and nuclear **(B)**. NF-κB in LPS-induced cells, LPS-pre-conditioned cells, and LPS-induced cells with caspase inhibitors from the cell lysates. The expressions were determined by western blot. Band densities were measured by densitometric analysis, and data for the expression of cytoplasmic and nuclear NF-κB was normalized to the control while the expression was expressed as fold change relative to beta-actin. Bars represent data from three independent experiments. NF-κB expression in LPS-induced cells, LPS-preconditioned cells, and LPS-induced cells with caspase inhibitors was compared to control. *p < 0.05 and **p < 0.01, as determined by one-way ANOVA with Bonferroni's correction.

To confirm the translocation of NF-κB from the immunofluorescence assay and evaluate the role of apoptosis, we inhibited apoptosis by using caspase inhibitors and measured the amount of NF-κB translocation from the cytosol to the nucleus in LPS-induced cells, LPS-pre-conditioned cells, and LPS-induced cells in the presence of synthetic non-specific caspase inhibitors, through western blot technique. In the LPS-induced cells, translocation of NF-κB from the cytoplasm into the nucleus (Figure 20) was observed and this was validated by the immunofluorescence results as shown in (Figure 13), however, the cells treated with caspase inhibitors showed significant downregulation of nuclear expression of NF-κB. The LPS-pre-conditioned cells had reduced expression of NF-κB in the nucleus in which most of the translocation confirming factor was retained in the cytoplasm, which was verified by the immunofluorescence staining method.

### Caspase Inhibition Downregulates p53 and Upregulates c-MYC and Hsp70 Expressions

**Figure 21:**
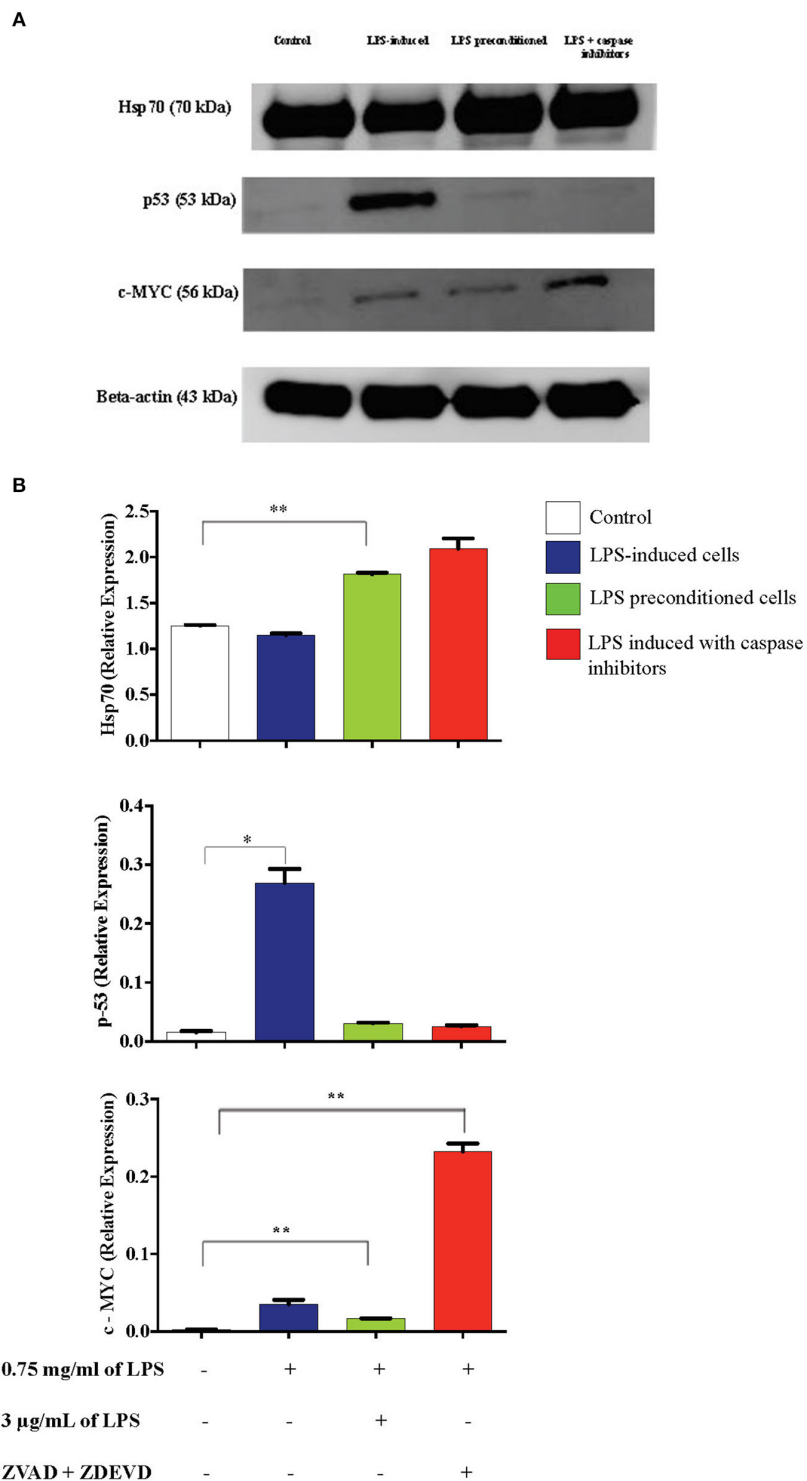
Expressions of Hsp70, p53, and c-MYC. **(A)** The expressions of Hsp70, p53, and c-MYC were determined in whole cell lysates by western blotting. Band densities were measured by densitometric analysis, and data were normalized to the control and expressed as fold change relative to beta-actin. **(B)** Bars represent data from three independent experiments. Hsp70, p53, and c-MYC expressions in LPS-induced cells, LPS-preconditioned cells, and LPS-induced cells with caspase inhibitors were compared to the control. *p < 0.05, **p < 0.01, as determined by one-way ANOVA with Bonferroni's correction.

**Figure 22:**
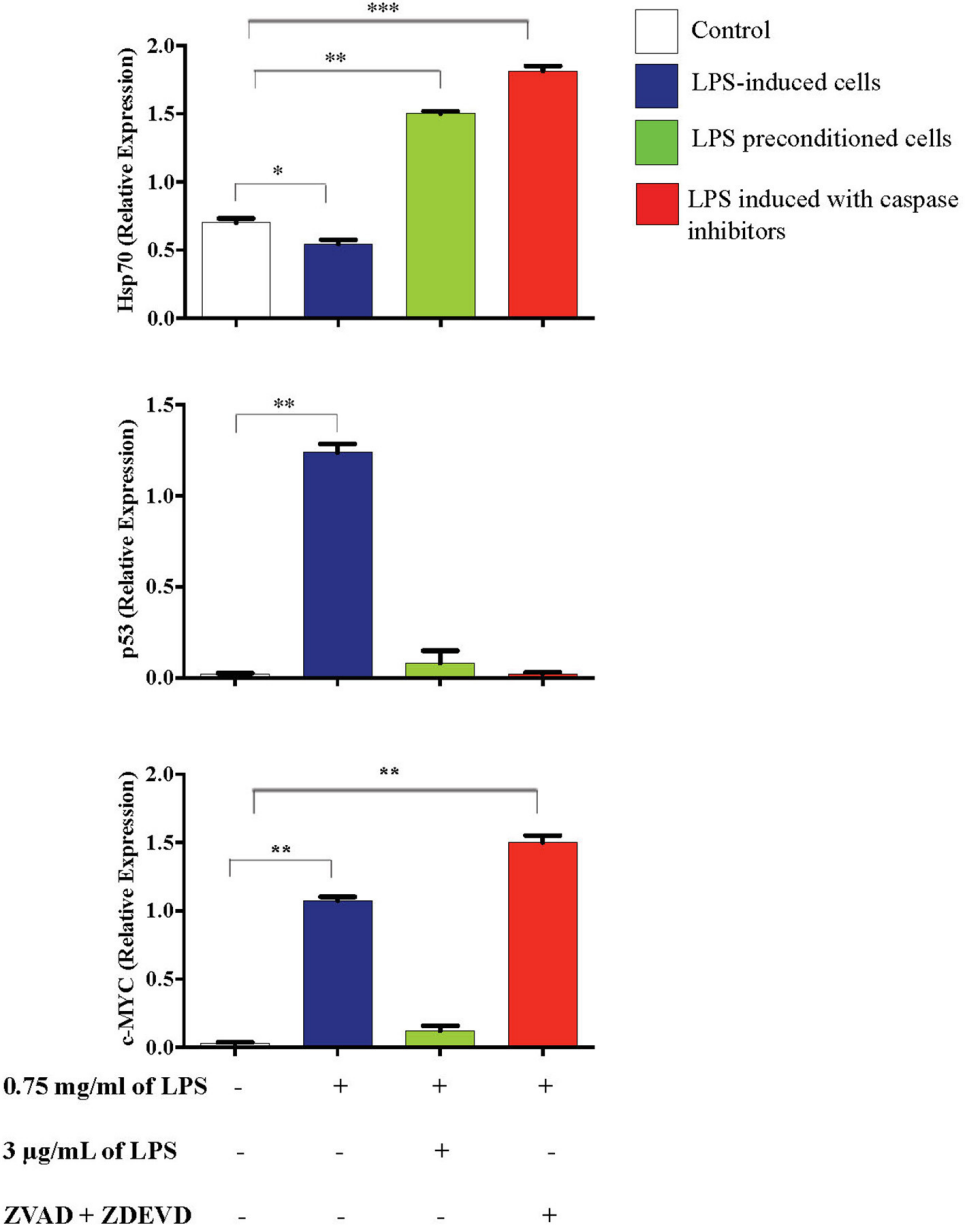
Expressions of Hsp70, p53, and c-MYC. The expressions of Hsp70, p53, and c-MYC were determined in whole cell lysates by ELISA. Bars represent data from three independent experiments. Hsp70, p53, and c-MYC expressions in LPS-induced cells, LPS-preconditioned cells, and LPS-induced cells with caspase inhibitors were compared to the control. *p < 0.05, **p < 0.01, ***p < 0.001 as determined by one-way ANOVA with Bonferroni's correction.

Inflammation is generally beneficial. However, extensive and prolonged inflammation is highly detrimental. One of the proteins involved in the cellular and molecular even of carcinogenesis is a tumor suppressor protein, p53, which is a key regulator of DNA repair, cell cycle progression, and apoptosis. Besides that, c-MYC is a transcription factor of proto-oncogenes and Hsp70 is a chaperone protein which is involved in cell cycle regulation and differentiation and is a hallmark protein in cancers. To probe this issue, we inhibited caspase activity in differentiated PC12 cells by using caspase inhibitors to exaggerate the inflammatory response and measured p53, c-MYC, and Hsp70 proteins by western blotting. As shown in Figure 21, Hsp70 was highly expressed in LPS-induced cells in the presence of synthetic non-specific caspase inhibitors and was also significantly higher in LPS-pre-conditioned cells compared to control cells. However, p53 expression was significantly higher in LPS-induced cells and was not altered in LPS-pre-conditioned cells and in LPS-induced cells with synthetic non-specific caspase inhibitors. In contrast, we observed significantly higher c-MYC expression in LPS-induced cells with synthetic non-specific caspase inhibitors and in LPS-pre-conditioned cells compared to control cells. For further validation, Hsp70, p53, and c-MYC proteins were measured using ELISA as shown in Figure 22, which had similar expressions as the western blot results.

### LPS Pre-conditioning Provides Cytoprotective Effect via TLR4/NF-κB Signaling Pathway

**Figure 23:**
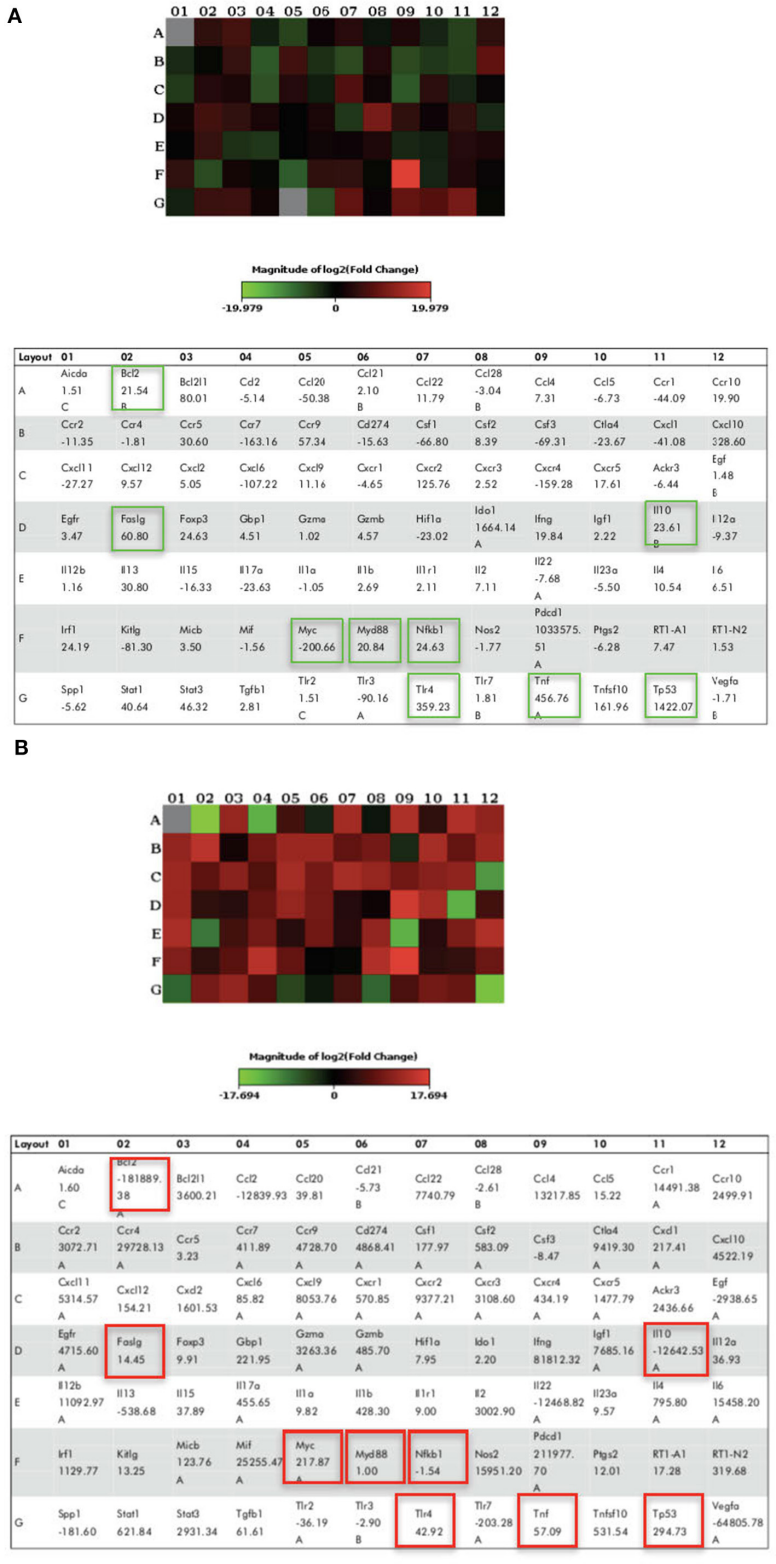
The Heat Map array provides visualization of fold changes in expression. **(A)** The fold changes in expression between LPS-induced and LPS-preconditioned cells (in green) for every gene in the array in the context of the array layout of three independent experiments. **(B)** The fold changes in expression between LPS-induced cells and LPS-induced cells with caspase inhibitors (in red) for every gene in the array in the context of the array layout of three independent experiments. The table provides the fold regulation data used for the map.

We demonstrated that LPS pre-conditioning protected the cells from undergoing apoptosis. Next, we investigated the potential mechanism associated with this response. We speculated that this might occur through the pre-activation of TLR4 signaling pathway which inhibits caspase-3/NF-κB signaling pathway. However, caspase-3/NF-κB signaling pathway inhibition may also promote oncogene expression, suggesting that this pathway might be involved in either protective or destructive effect. A 96-well plate RT^2^ Profiler PCR array was performed for LPS-induced cells, LPS-pre-conditioned cells and LPS-induced cells with synthetic non-specific caspase inhibitors. Collectively, the gene expressions (in green and red box) elucidated the possible molecular signaling pathway and we concluded that LPS pre-conditioning exerts a protective effect via TLR4/caspase-3/NF-κB signaling pathway (Figure 23).

## Discussion

The present study evaluated the concentration and time period which confer cytoprotective potential of LPS pre-conditioning against apoptosis in differentiated PC12 cells. We found that pre-conditioning with 3 μg/mL for 12 h protected the differentiated PC12 cells against subsequent exposure to 0.75 mg/mL apoptotic concentration of LPS, which can be explained as endotoxin tolerance. To date, our study is the first preliminary study to be established as an *in vitro* model of LPS pre-conditioning in differentiated PC12 cells which show that LPS pre-conditioning causes significant protection against subsequent exposure to apoptotic concentration of LPS.

Before starting the experiment, optimization of the concentration and time was conducted as a preliminary study to determine the optimal LPS concentration and time. Therefore, in our study, differentiated PC12 cells were exposed to 50, 25, 12.5, 6.25, 3.125, 1.563, and 0.7815 μg/mL LPS for 12, 24, 48, and 72 h. Even though 1.563 and 0.7815 μg/mL were lower than 3 μg/mL and maintained the cell viability at 100%, we chose the highest concentration among these three concentrations. As such, the highest low concentration of LPS which exhibited cell viability of 100% (3 μg/mL) was chosen for subsequent study.

Furthermore, the inhibitory concentration (IC_50_) was determined with LPS concentrations of 1, 0.5, 0.25, and 0.125 mg/mL for 12, 24, 48, and 72 h. We found that 0.75 mg/mL for 12 h was our IC_50_, which inhibited 50% of the cell viability (data not shown)_._ However, there is no direct evidence to evaluate whether this concentration is suitable as the apoptotic concentration. Therefore, we modified and optimized the concentration and time point. The findings for pre-treatment of PC12 cells with 3, 6, 9, and 12 μg/mL LPS for 12, 24, 48, and 72 h and subsequent exposure with 0.75 mg/mL LPS for 12, 24, 48, and 72 h confirmed that pre-conditioning with 3 μg/mL LPS protected the cells against apoptosis. Surprisingly, based on our MTT results, the pre-conditioning concentration of 3 μg/mL LPS at 12 h significantly inhibited apoptosis against subsequent LPS challenge (0.75 mg/mL LPS for 12 h) and maintained cell viability, indicating that there was no significant cytotoxicity in the pre-conditioned cells. In contrast, when the cells were induced with 6, 9, and 12 μg/mL LPS concentration, the cytoprotective effect disappeared and the treatment became deleterious. Even though the cells were pre-conditioned with low LPS concentration, subsequent LPS challenge at apoptotic concentration for longer hours became toxic to the cells and caused them to lose viability.

This is further confirmed by assessing the morphology of the cells where the LPS-pre-conditioned cells displayed normal cellular structures as the control. Moreover, the cell cycle analysis confirmed that at the G_1_ phase, the cells progressively lost viability, died over time, and did not enter the cell cycle. Further analysis with Annexin V-FITC also confirmed that LPS-pre-conditioned cells with 3 μg/mL offered cytoprotective effect where the cells were shown to be populated in the lower left quadrant, similar to the control. Therefore, a single low concentration of 3 μg/mL LPS conferred cytoprotection against LPS-induced apoptosis.

This is supported by a recent novel finding which showed a single low dose of 0.2 mg/kg LPS pre-conditioning successfully prevented neurodegeneration by reducing post-injury gliosis response near the corpus callosum. The authors found that a low dose of LPS pre-conditioning is protective in a closed-head model of diffuse axonal injury. They also observed that a low dose of LPS pre-conditioning regulated glial activity, which protected against diffuse axonal injury and recovered completely by time of injury (Turner et al., 2017).

In addition, Hickey et al. (2011) reported that a low dose of LPS pre-conditioning provided a delayed protection against brain injuries, which is related to hypothermic circulatory arrest in piglet model. They pre-conditioned with LPS at 20 mg/kg for 3 days before the brain injury. They found that LPS pre-conditioning conferred global cerebral protection by reducing injury in the cortex, basal ganglia, and hippocampus.

Rosenzweig et al. (2007) found that mice treated with various doses of LPS between 0.05 and 0.2 mg/kg showed significant protection compared to saline-treated controls. They also pre-conditioned mice with LPS for different time intervals before middle cerebral artery occlusion (MCAO) and stroke outcome. Interestingly, they observed LPS-induced neuroprotection within a day. The authors reported the specific dose range and time frame of LPS pre-conditioning in mice (Rosenzweig et al., 2007). In line with that, the study supports our findings that LPS pre-conditioning provides cytoprotection against apoptosis in subsequent challenges, depending on concentration and time. Therefore, these studies provide guidelines and literatures for our study where various concentration and time points should be considered.

The LPS pre-conditioning effect provides cytoprotection depending on the concentration and time. It should be taken into consideration that in animal models, a single systemic dose of 0.05–1 mg/kg is normally administered but the dose is highly toxic for humans. However, in *in vitro* models, the low dose of 1 mg/mL LPS pre-treatment for 24 h protected cortisol neurons from oxygen/glucose deprivation (OGD)-induced cell death (Lastres-Becker et al., 2006). Therefore, the doses vary according to the cells and models.

Another factor to be considered in conferring protection apart from the concentration of the stimulus is the time period. In our present study, the initial time point to provoke the inflammatory cascade was 12 h. The second pre-conditioning stimulus to provide protection, referred to as the second window of protection (SWOP), was also 12 h. The period which confers protection occurs between 12 and 72 h after the first stimuli. The time period within SWOP that provides cytoprotection could be due to protein synthesis and post-translational modifications. Thus, it could be explained that LPS provokes the inflammatory response, which triggers the cell surface receptors and initiates the signaling cascade of events (Marber et al., 1993; Yellon and Downey, 2003). Perhaps, the time frame for induction of tolerance in LPS-induced cells is required for the secretion of cytokines and chemokines (Lapidot and Petit, 2002). Other than that, both LPS-pre-conditioned and LPS-induced apoptotic cells share the same signaling pathway by promoting or inhibiting particular proteins. For that reason, the specificity of proteins is studied and explained later. Together, these results confirmed that the pre-conditioning concentration of 3 μg/mL LPS for 12 h was able to offer protection against apoptosis in response to subsequent exposure to 0.75 mg/mL LPS for 12 h. However, the concentration, time window of tolerance, and pre-conditioning phenomenon will be different with primary stimuli, secondary insults, and window period, and thus might give different results with different models.

In our research, we were interested to understand the NF-κB signaling pathway in LPS-pre-conditioned and LPS-induced cells. Multiple factors contribute to neuroprotection following LPS-induced pre-conditioning. Translocation of NF-κB induces the expression of pro-inflammatory cytokines and in contrast, the inhibition of NF-κB provides neuroprotection.

In our study, we showed that in LPS-induced cells, NF-κB was translocated from the cytosol into the nucleus and this was then associated with the induction of various inflammatory genes. NF-κB represents a family of inducible transcription factors and regulates a large array of genes involved in different processes of the immune and inflammatory responses (Oeckinghaus and Ghosh, 2009; Liu et al., 2017). Therefore, we detected the expression of NF-κB activation via the reduction of IκB in our current study.

Although we did not investigate this at the receptor level, LPS is a well-known ligand for TLR4 (Park and Lee, 2013) and we speculate that LPS binding to TLR4 receptor triggers the activation of a central adapter protein for the TLR family, MyD88 (Troutman et al., 2012). This initiates the activation of the extrinsic pathway through caspase 8, which then cleaves and activates effector caspase 3/7, leading to apoptosis. We showed herein that this was associated with the secretion of various inflammatory cytokines and chemokines including TNF-α, which is a potent inducer of LPS (Kikkawa et al., 1998). The cytoprotection afforded by TNF-α in LPS-pre-conditioned cells, and in contrast, detrimental effects shown in LPS-induced apoptotic cells, prove the dose-related protective and deleterious effects. Remarkably, the effect of TNF-α depends upon which receptor, TNFR 1 or TNFR 2, is being activated. TNF-α may promote the activation of two different pathways: one of the pathways could cause cell death via p55 TNF receptor (TNFR 1), while the other pathway results in neuronal survival through p75 TNF receptor (TNFR 2), depending on the expression of a wide array of genes that it induces, which are genes involved in either cell death or cell survival (Papadakis and Targan, 2000; Aggarwal, 2003; Chen and Palmer, 2013). Besides, the level of activation of TNF-α may be important in determining the deleterious or beneficial effect contributing to the dose-related protective effect of LPS in the brain (Bordet et al., 2000).

We also showed that inhibition of NF-κB translocation results in secretion of various anti-inflammatory cytokines such as IL-1Ra, IL-10, CNTF, and IL-13. Pre-exposure to low concentration of LPS could confer a protective state against cellular apoptosis following subsequent stimulation with LPS at higher concentration, suggesting a role for TLR-4 pre-activation in the signaling pathway of LPS-induced cytoprotection (Vartanian et al., 2011). This result suggests that anti-inflammatory cytokines are potential molecular targets, although future study is warranted to clarify their role and the underlying mechanism regulating their expression and/or secretion in pre-conditioned cells.

Moreover, our results demonstrate that NF-κB is present in the cytoplasm in an inactive form and gets translocated into the nucleus upon LPS stimulation. Then, the inhibitory kappa B (IκB) gets phosphorylated by inhibitory kappa B kinase, causing the activation of inflammatory gene transcription (Shih et al., 2015). We postulated that LPS induces inflammation via TLR4/NF-κB signaling pathway, which induces the secretion of cytokines and chemokines and apoptosis mechanism via extrinsic pathway. Indeed, the apoptosis mechanism mediated via death receptor which recruits caspase 8 is a critical mediator for the extrinsic pathway, which in turn activates caspase 3/7 and finally results in apoptosis.

In addition, ROS and NO also activate NF-κB signaling pathway, which leads to the upregulation of pro-inflammatory cytokine expression in LPS-induced cells (Yamamoto and Gaynor, 2001). In line with that, we showed that the secretion of ROS and NO upregulated Bax protein, a pro-apoptotic protein which contributes to neuroinflammation (Block et al., 2007; Glass et al., 2010; Lull and Block, 2010).

The excessive production of the inflammatory mediators may cause chronic neuroinflammatory diseases such as neurodegenerative diseases including Alzheimer's disease, Parkinson's disease, and stroke (Glass et al., 2010). However, our results show that LPS-pre-conditioned cells attenuated the production of inflammatory mediators ROS and NO against LPS-induced apoptosis. Yet, more efforts are needed to understand the mentioned mechanism.

LPS-induced cells activate the death ligand pathway and ultimately the mitochondrial apoptotic pathway, which leads to neuroinflammation and interferes with the Bcl-2 family (Reed, 2002; Wennersten et al., 2003). Bax has a distinct homology with Bcl-2, but the function of Bax is contradicted by Bcl-2 anti-apoptotic members. Indeed, Bax causes mitochondrial outer membrane permeabilization (MOMP) by destabilizing the lipid bilayer, creating pores or interacting channels. Several studies have indicated that upregulation of Bax and downregulation of Bcl-2 trigger cytochrome c release from the mitochondria into the cytosol, leading to apoptosis and eventually may contribute to the pathogenesis of neurodegenerative diseases (Savory et al., 2003; Lin and Beal, 2006). The cytochrome c activates executioner caspase-3, which will be discussed. We believe the protection was correlative with upregulation Bcl-2. However, it is not known whether more apoptosis pathways and molecules are involved in LPS-induced cross-tolerance in neuroinflammation and future studies are needed to address this issue. This LPS-induced apoptosis model can activate inflammatory cascade *in vitro*, upregulating pro-inflammatory cytokines, inflammatory mediators, and pro-apoptotic proteins. In contrast, LPS-pre-conditioned cells secrete anti-inflammatory cytokines and anti-apoptotic proteins to attenuate the detrimental process.

We speculated that low concentration of the LPS binds to TLR4, which reprograms TLR4 signaling in response to a subsequent apoptotic concentration of LPS. Interestingly, the cells which were re-exposed to LPS were characterized by a reduction in pro-inflammatory cytokines; in other words, pre-conditioned cells were protected and have increased cell resistance. Therefore, LPS pre-conditioning can be protective with anti-inflammatory cytokines such as IL-1Ra, IL-10, CNTF, and IL-13 being secreted as feedback inhibitors to terminate the LPS response (Shpargel et al., 2008; Vartanian et al., 2011). Among the anti-inflammatory cytokines, IL-10 has been suggested as the principal mediator of endotoxin tolerance and can be protective against inflammatory damage. Besides, a study by Nayak et al. (2009) supported our study, where a high expression level of IL-10 was observed among stroke-recovering patients. However, the exact mechanism of pre-conditioning remains to be explored.

Furthermore, the study by Lin et al. (2009) found that low dose of LPS pre-conditioning in neonatal rats suppressed hypoxic ischemia-induced neuroinflammation and conferred neuroprotection against behavioral and pathologic abnormalities. Therefore, their findings are parallel to our study where they elucidate the protective mechanism of LPS pre-conditioning, even though the experimental settings were different.

In addition, Kumral et al. (2012) presented that their first study to evaluate the role of LPS pre-conditioning-induced white matter in injury model. The author also posited that the most critical point about pre-conditioning is the period between the non-injurious dose and the lethal dose. They also suggested that inflammatory stimulation by LPS induces antioxidant enzyme expression in the immature brain.

Our main findings of our investigation showed LPS pre-stimulation may protect the cells from undergoing apoptosis. This may occur via the extrinsic pathway through caspase 8 activation, which, in turn, activates effector caspase 3/7. We speculated that this might occur through the pre-activation of TLR4 signaling pathway leading to the inhibition of caspase-3/NF-κB pathway. In addition, LPS pre-conditioning may also contribute to the cytoprotective effect against apoptosis through increased production of anti-inflammatory cytokines and expression of anti-apoptotic protein.

Therefore, this study provides a basis for future research to better understand the molecular mechanism underlying LPS pre-conditioning/TLR4 pre-activation and its role in immune tolerance in neuronal environment. It is speculated that the low dose of LPS suppresses caspase pathway and increases the anti-apoptotic protein, production which is correlated with neuroprotection and endotoxin tolerance by suppressing neuroinflammatory cascade.

On the other hand, chronic neuroinflammation is the pathogenesis of many other neurodegenerative diseases. The CNS has been experimentally and clinically proven to modulate the inflammatory response to attenuate damages and confer neuroprotection (Lucas et al., 2006). There are various evidence supporting that glial cells in CNS play dual role as they both could have neurotoxic or neuroprotective effects. Consequently, pro-inflammatory and anti-inflammatory cytokines being secreted via multiple signaling pathways and their activities could also be overlapping (Block et al., 2007). Therefore, our study elucidates the signaling pathway involved in detrimental and cytoprotective mechanisms.

To achieve our objective of elucidating the signaling pathway that provides cytoprotection and induces oncogene expression, we performed gene expression array. Among the 84 genes, we were interested to identify the genes involved in the downstream signaling pathway which regulates the receptors, adaptor proteins, transcription factor, inflammatory cytokines, tumor suppressor genes, and oncogene. The studied genes were TLR4, MyD88, NF-κB, TNF, Faslg, Bcl-2, IL-10, p53, and c-MYC. We found that LPS binds to TLR4, which mediates the downstream signaling pathway.

Although previous studies have shown and mimicked inflammatory pathway, the mechanism is not fully studied. Combining all the theories, LPS has the ability to bind to PAMPs especially TLR4/MyD88 (Kawai et al., 2001; Kawai and Akira, 2010). This induces the downstream signaling pathway, which leads to cytokine and chemokine production. Moreover, the translocation of NF-κB into the nucleus triggers the transcription of genes involved in inflammation. Therefore, TLR4 can promote the production of inflammatory mediators, especially TNF-α, which have been implicated in neuroinflammation and apoptosis. We postulated that LPS induces inflammation via TLR4/MyD88/NF-κB signaling pathway, which induces the secretion of cytokines and chemokines and apoptosis mechanism via the extrinsic pathway (Rahimifard et al., 2017). Indeed, the apoptosis mechanism mediated via death receptor which recruits caspase-8 is a critical mediator for the extrinsic pathway, which in turn activates caspase 3/7 and finally results in apoptosis (Danial and Korsmeyer, 2004).

LPS pre-conditioning is a well-established method for immune modification, which has been shown to attenuate neuronal damages in animal models. Kigerl et al. (2009) found that the M1 phenotype (activated microglia) is activated by TLR or IFN-γ, which has a toxic effect. In contrast, M2 phenotype is activated by 1L-4, IL-10, and IL-13 which has regenerative effect *in vitro* and *in vivo*. Furthermore, Yamamoto et al. (2003) discovered that TLR4, the receptor for LPS, binds to MyD88 and TRIF, the two main adapter proteins, and activates MyD88–NF-κB signaling pathway and TRIF-(Interferon Regulatory Factor) IRF pathway and this is supported by the findings of Honda et al. (Yamamoto et al., 2003; Honda and Taniguchi, 2006). Samanta et al. (2008) and Biswas et al. (2007) reported that IL-10, an anti-inflammatory cytokine, is regulated by IRF-3. Therefore, collectively we could conclude that LPS pre-conditioning induces LPS/TLR4 signaling pathway via TRIF-IRF pathway and secretes IL-10 cytokines.

Vartanian et al. (2011) showed that LPS pre-conditioning suppressed NF-κB activity and enhanced IRF-3 activity and anti-inflammatory gene expression in an *in vivo* model of ischemic brain. In fact, they provided the first evidence that LPS pre-conditioning stems from TRIF signaling, the cascade which is associated with IRF3 activation via MyD88 independent signaling pathway.

Recently, much attention has been given to therapeutic strategies for neuroprotection which are targeted to inhibit the deleterious effects. Therefore, LPS pre-conditioning was introduced, which has been shown to have neuroprotective effects in many studies (Dirnagl et al., 2009). Our present study findings are consistent with those of previous studies. Our results demonstrate that administration of a low concentration of LPS is effective in reducing the consequences of apoptotic responses via TLR4/MyD88 signaling in the extrinsic apoptotic pathway. As a downstream signal, the anti-inflammatory and anti-apoptotic cytokine Bcl-2 is implicated to attenuate the pro-inflammatory cytokines and pro-apoptotic protein Bax (Bolondi, 2012). Moreover, Bcl-2 provides protection through the inhibition of cytochrome c release from mitochondria, thus preventing the activation of apoptotic pathways (Ouyang and Giffard, 2004). Even though Bcl-2 acts as an anti-apoptotic protein, it is also known as an oncoprotein. However, overexpression of Bcl-2 alone is not significant to cause cancer (Coultas and Strasser, 2003).

LPS pre-conditioning reduces the expression of caspase-3, an apoptotic marker which is driven as inhibition of caspase pathway and upregulates anti-apoptotic protein (Sun et al., 2015). Genomic expression patterns revealed that LPS pre-conditioning leads to protection against apoptosis via a process called “reprogramming.” This could be explained as by the exposure to low concentration of LPS, where the cell modulates and secretes anti-inflammatory cytokines upon the higher exposure LPS concentration. This protective state known as tolerance and it is not well understood, although emerging evidence suggests that modulation of inflammatory response plays a role (Gidday, 2006; Rosenzweig et al., 2007).

Besides the TLR4/MyD88 signaling pathway, Faslg and TNF were also expressed in LPS-induced, LPS-pre-conditioned, and LPS-induced with caspase inhibitors cells. Faslg and TNF are key pro-apoptotic molecules which are expressed by inflammatory cells. Both proteins could induce inflammation through the production of various cytokines and chemokines (Hallenbeck, 2002).

FasL is a member of TNF superfamily of cytokines. The extrinsic pathway is triggered by ligation of the death receptor such as FasL and TNF and mediated by direct activation of caspases (Wajant, 2002). Even though genetic and environmental factors are responsible for various disorders, they have a shared mechanism of intrinsic and extrinsic pathway of neuronal apoptosis. Unfortunately, little is known about the intrinsic apoptosis mechanism in CNS. FasL is expressed in normal CNS and significantly elevated in inflamed and degenerated brains. Perhaps, FasL should be considered as a double-edged sword in the CNS, which maintains the immune regulation and induces neuronal cell death and inflammation in brain diseases (Choi and Benveniste, 2004; Tian et al., 2009).

From our findings, TNF and FasL were involved to induce the extrinsic pathway. It requires the recruitment of adaptor proteins such as FADD and TRADD (not measured in our study) to induce and mediate the caspase-8. Consequently, caspase-8 cleaves the Bcl-2 family and subsequently activates the intrinsic pathway of apoptosis (Kantari and Walczak, 2011). Therefore, the extrinsic apoptosis pathway is cross-linked with the intrinsic apoptosis pathway. Unfortunately, we did not measure TNFR family in our study to understand the depth of the mechanism. As a result, we could not clearly explain the protective mechanism that involves TNFR 2. Recent finding suggests that TNFR 2 plays an essential role in cell survival, but the mechanism is far more elusive in revealing its signal transduction pathways (Marchetti et al., 2004).

The molecular and cellular events that characterize apoptosis require the activation of caspases which mediate cell death. However, during the development, the activity of caspases is taken care by Inhibitors of Apoptosis Proteins (IAP), which allows the survival of cells. Importantly, initiation of apoptosis requires the inhibition of the IAPs, which can be triggered by various inducing agents (Fulda and Vucic, 2012). Therefore, in the present study, we inhibited caspase activity using a pan-caspase inhibitor (Z-VAD-FMK) and selective caspase-3 inhibitor (Z-DEVD-FMK) to investigate the role of caspase-3 in inflammation and oncogene expressions (Abdi et al., 2011). We found that inhibiting caspase-3 reduced the expression of nuclear NF-κB expression. Thus, it could be postulated that caspase-3 is one of the important molecules in NF-κB activation. Caspase-3 is the main executioner caspase in apoptotic cell death and plays an important role in apoptosis, contributing to neuroinflammation and neurodegeneration (Mukherjee and Pasinetti, 2001; Krady et al., 2005). Besides, caspase-3 is an important molecule for NF-κB activation; hence, inhibiting caspase-3 prevents the degradation of IκB (inhibitor of kappa B), leaving NF-κB in its inactivated form in the cytosol (Xu et al., 2006).

Therefore, the inactivated NF-κB in the cytosol resulted in its reduction in the nucleus. Furthermore, it could be explained that apoptosis and NF-κB activation regulate the apoptosis mechanism in neuroinflammation, where NF-κB acts as a pro-apoptotic protein (Milani et al., 2003). Collectively, we speculated that there is a link in mediating apoptosis via caspase-3 and NF-κB regulation. Inhibiting caspases by caspase inhibitors could result in the inhibition of apoptosis, an increase in the production of oncogenes, and a downregulation of tumor suppressor gene (Indran et al., 2011).

Following caspase inhibition, we measured total caspase-3, p53, c-MYC, and Hsp70 expressions. In addition, we also measured NF-κB expression in the cytoplasm and nucleus of the cells. Our results showed that caspase inhibition inhibited NF-κB translocation and activated c-MYC and Hsp70 but downregulated p53. p53 is a tumor suppressor protein that regulates multiple signaling pathways triggered by diverse cellular stresses, including DNA damage and oncogenic events (Jebelli et al., 2014).

In addition, Hsp70 is a chaperone protein, found most abundantly in cells and is a major stress-induced cytoplasmic chaperone. In contrast, it should be mentioned that overexpression of Hsp70 plays a role in brain ischemia and ischemic tolerance, which can exert beneficial effects in neurodegenerative diseases (Turturici et al., 2011). In *in vivo* models of pre-conditioning, it has been reported that Hsp70 is highly expressed and many studies have implicated that Hsp70 is an important player in the pre-conditioning process (Yeh et al., 2010). Besides, our results show that caspase inhibition in LPS-induced cells inhibited the translocation of NF-κB and resulted in the overexpression of Hsp70. Indeed, researchers have also found that Hsp70 is highly expressed in malignant tumors which are linked to cancer (Garrido et al., 2003; Chen et al., 2006). However, a study by Sabirzhanov et al. (2012) suggested that Hsp70 expression increased through the pre-conditioning method is a promising therapeutic approach for treatment of neurodegenerative diseases.

On the other hand, c-MYC is a transcription factor and proto-oncogene which is highly regulated in cancers. It is also involved in cell growth, cell division, metabolism, and differentiation (Dang et al., 2009). Dysregulation of c-MYC causes cancer when caspase activity is disrupted. When the apoptosis pathway is disrupted by caspase inhibitors, the expression of c-MYC promotes tumorigenesis (Fabregat et al., 2007). Therefore, we conclude from the findings that caspase inhibition increases the expression of Hsp70 and c-MYC proteins and downregulates p53, which suggests that caspase inhibition prevents NF-κB activity by blocking the NF-κB translocation from cytosol to nucleus. In addition, there are a large number of studies suggesting that cytokines and chemokines are candidates that link inflammation and oncogene expression (Mantovani et al., 2008; Mantovani, 2010).

Inhibition of apoptosis mechanism could be linked to chronic inflammation and oncogene expressions. In line with that, many studies have demonstrated the crosslink between chronic inflammation and cancer. Inhibiting the apoptosis mechanism can result in chronic inflammatory diseases, autoimmune diseases, and cancers. This is because the inhibition cell death will lead to excessive cellular proliferation, resulting in malignant tumors and expression of oncogenes (Igney and Krammer, 2002; Okada and Mak, 2004).

Besides, genes that regulate the cell cycle alter the molecular mechanisms, which create some confusion as to their significance in the genesis and progression of cancer by activating oncogenes and inactivating tumor suppressor genes. In these scenario, we could conclude that there is a crosslink between chronic inflammation and oncogene expressions (Evan and Vousden, 2001; Sherr, 2004; Colotta et al., 2009). However, further studies are needed to understand the mechanism of genetic complexity in the downstream molecular signaling pathway.

Therefore, our study suggests that LPS pre-conditioning demonstrates cytoprotective effect by suppressing caspase-3 and NF-κB activity, leading to inhibition of pro-inflammatory cytokines and chemokine production in pre-conditioned cells. This suggests that LPS pre-conditioning has a protective effect by promoting endotoxin tolerance and suppressing neuroinflammatory cascade (Yokobori et al., 2013; Wang et al., 2015). From our study, we discovered that the inhibition of caspase-3/NF-κB signaling pathway could have a dual role of protecting the cells against apoptosis or promoting oncogene expression.

Finally, our study may helpful to set a platform for more in-depth studies about the cytoprotective mechanism conferred by LPS pre-conditioning against LPS-induced apoptosis in differentiated PC12 cells. Our study also sheds light on the mechanism of neuroprotection and neuroinflammation pathogenesis and the molecules involved in downstream signaling pathways. Our preliminary study could resolve and explain exceptions related to neuroprotection provided by LPS pre-conditioning via anti-inflammatory cytokines and anti-apoptotic protein in future *in vivo* studies. The experimental approach of LPS pre-conditioning has potential clinical applications by identifying the regenerative mechanism and pathways. Collectively, pre-conditioning-based strategies might have potential advantage of modulating the immune cascades to induce protection against neuropathology diseases.

Currently, stem cell transplant is a potential regenerative therapy for many diseases particularly for neurodegenerative diseases. Stem cells and progenitor cells provide trophic supports and regulate regenerative mechanisms. Many different stem cells are under pre-clinical and clinical investigations for various disorders. Yet, many problems remain unsolved such as ethical concerns, cell-specific differentiation, homing into lesion sites, and neural network complications. However, recent findings have shown that combining pre-conditioning with stem cell therapy provides the opportunity of clinical applications of hypoxic pre-conditioning. Exposure of stem cells or progenitor cells with sublethal hypoxia or any other pre-conditioning stimulants have been shown to increase the cells resistance against multiple injurious insults after transplantation. The pre-conditioned stem cells and progenitor cells showed improvement in cell survival, neuronal differentiation along with the secretion of trophic support, and enhancement of the homing to the lesion site. Therefore, the combination of pre-conditioning in stem cells will definitely attract more attention in stem cell and regenerative translational research, which will broaden the possibility for clinical applications (Hu et al., 2008; Theus et al., 2008; Ogle et al., 2009; Yu et al., 2013).

**Figure 24:**
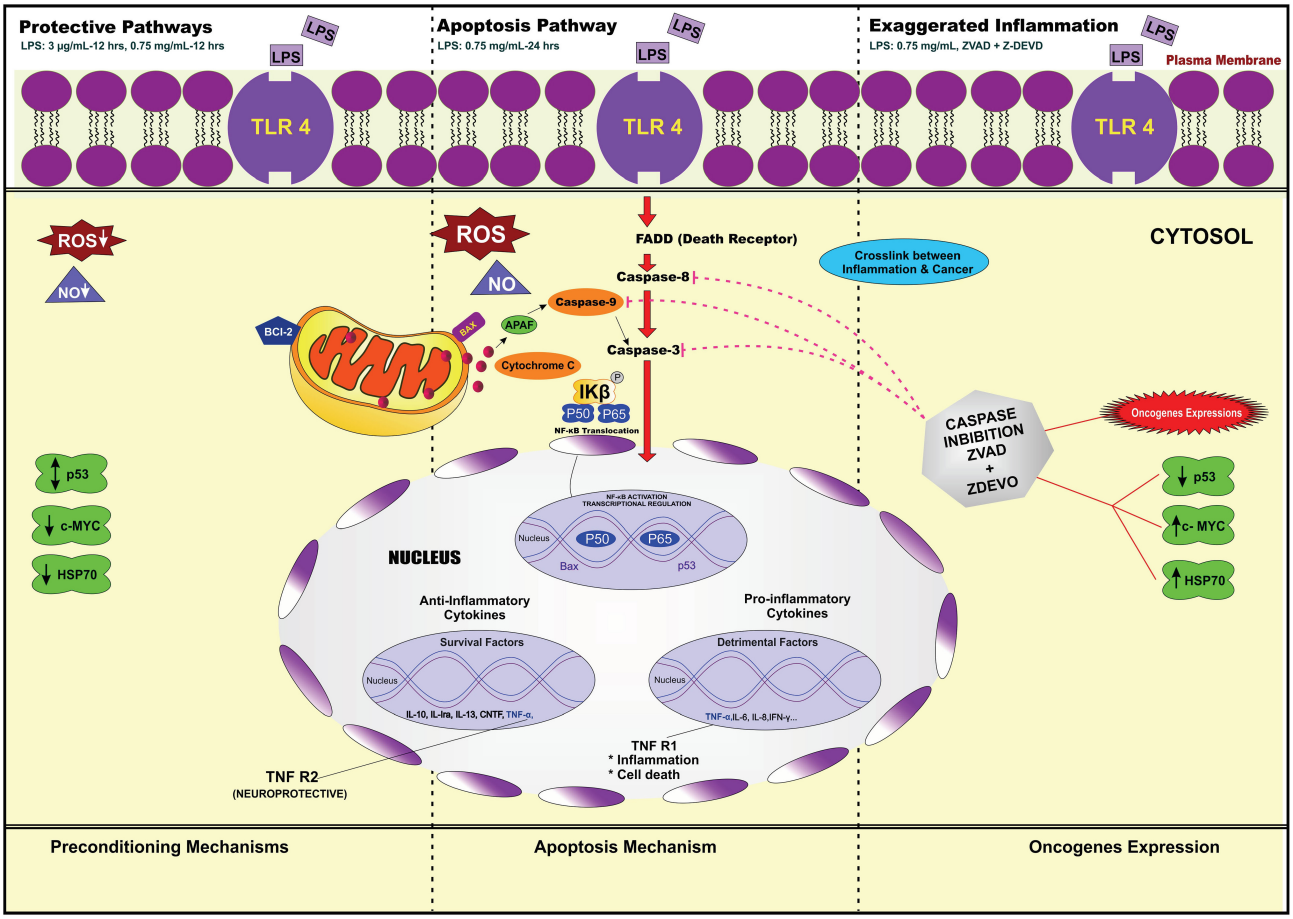
Overview of the molecular signaling pathway. It is involved in LPS-preconditioned cells, LPS-induced cells, and LPS-induced cells incubated with caspase inhibitors via TLR4/caspase-3/NF-κB signaling pathway in differentiated PC12 cells. Transcriptional factor NF-κB and caspase-3 are associated with cytoprotection, inflammation, and activation of oncogene expressions.

In conclusion, we found that LPS pre-conditioning has a cytoprotective effect on PC12 cell lines against LPS-induced apoptosis. The present study provides new insights into the effect of LPS-induced caspase inhibition in chronic neuroinflammation *in vitro* as shown in Figure 24. From this study, we suggest that the modulation of the immune system through LPS pre-conditioning could be widely applicable to many other neurodegenerative diseases that involve neuroinflammation. However, further studies are required before LPS treatment in clinical settings can be practically applied.

## Conclusion

In conclusion, the pre-conditioning technique could be used to identify the proteins and genes involved in the LPS-signaling pathway and their functional roles in offering cytoprotective response. The technique offers an unbiased approach to induce robust protection against subsequent lethal injuries. This dataset could be used as a reference in future studies to better understand the mechanism of neuroprotection and neuroinflammation and may also help in finding new specific molecular signaling pathways that could promote neuroprotection or reverse/inhibit neuroinflammation.

## Data Availability Statement

The raw data supporting the conclusions of this article will be made available by the authors, without undue reservation.

## Author Contributions

PGS performed experiments, collected data, conceived of the idea for the paper, and wrote the manuscript. ZC provided inputs on data analysis. ZAI, ZM, and AA provided critical guidance and worked on the manuscript. All authors approved of the final manuscript.

## Conflict of Interest

The authors declare that the research was conducted in the absence of any commercial or financial relationships that could be construed as a potential conflict of interest.
